# An optimized protocol for metabolic measurement in 3D tumor spheroids derived from primary and established glioblastoma cells

**DOI:** 10.1371/journal.pone.0347569

**Published:** 2026-04-24

**Authors:** Samiya Al-Robaiy, Urszula Hohmann, Andreas Simm, Faramarz Dehghani, Julian Prell, Christian Strauss, Tim Hohmann

**Affiliations:** 1 Center for Medical Research (ZMG), Martin Luther University Halle-Wittenberg, Medical Faculty Halle, Halle, Germany; 2 Department of Anatomy and Cell Biology, Medical Faculty, Martin Luther University Halle-Wittenberg, Halle (Saale), Germany; 3 Clinic for Heart Surgery (UMH), Medical Faculty Halle, Martin-Luther-University Halle-Wittenberg, Halle (Saale), Germany; 4 Department of Neurosurgery, University Hospital Halle (Saale), Halle, Germany; Universidad Nacional Autonoma de Mexico, MEXICO

## Abstract

Tumors are characterized by a multitude of genetic and epigenetic alterations, including a deregulation of the metabolism, driving migration and infiltration. To mimic the energetic landscape of *in vivo* tumors, 3D models surpass traditional 2D cultures, by introducing regions of different nutrient and oxygen supply. Yet, the analysis of metabolic processes in 3D cultures, including the mitochondrial answer and extracellular fluxes is more challenging. The extracellular flux analyzer is a powerful tool for investigating cellular metabolism, offering valuable insights that can drive advancements in biomedical research, but protocols for analysis of 3D cultures are sparse. Here, we present a protocol for optimized extracellular flux analysis, starting from the choice of the 3D culture model, dependencies on 3D culture size and testing multiple normalization approaches for two different glioblastoma and two primary cell lines. It was demonstrated that our approach was feasible for different glioblastoma cell lines, showing cell type and spheroid size dependent responses to metabolic challenges. In addition, normalization approaches using essentially 2D characteristics of spheroids were found insufficient to account for different spheroid sizes and cell lines. The data showed that using bio-printed spheroids with magnetic beads, combined with normalization to the median values of an experiment and the initially seeded cell number, delivered the most reliable results. Thus, we provided an approach that enables a straightforward and reproducible generation of 3D cell cultures and offer strategies to optimize metabolic measurements within these cultures.

## Introduction

Malignant tumors in general are characterized by their capabilities for migration, unlimited proliferation etc. [1,2]. Notably, these processes are in high demand of energy. Consequently, understanding the energetic landscape of tumors or its modification are promising and interesting targets in tumor research. To study tumor biology a multitude of *in vitro* models were used, with classical 2D cultures being still very prominent today. Among others, 2D cultures fail to mimic certain key features of *in vivo* tumors, including their heterogeneity, nutrient and treatment gradients [3,4]. Thus, more complex 3D models were developed and used trying to include the abovementioned parameters. One of the most widely used 3D tumor models are tumor spheroids, which have more complex cell-cell contacts and nutrient gradients comparable to those found in tumors and thus differ metabolically from 2D cultures [5–7].

Spheroids are aggregates of cells and represent avascular tumor nodules or micro-metastases [6]. Tumors *in vivo* show several biochemical gradients (i.e., oxygen, metabolites) and different physical properties which substantially impact cell behavior, resulting in a heterogeneous response to treatment [8]. To model different levels of heterogeneity inside a tumor spheroid, its dimensions can be modified. Large spheroids (> 500 μm in diameter) are composed of several specialized areas and layers where cells display different phenotypic, functional, and metabolic behaviors. They contain a proliferative layer at the spheroid edge, an intermediate zone composed of quiescent and/or senescent cells and an apoptotic and necrotic core resulting from the differential supply of nutrients and oxygen in these areas [3,9]. This particular organization strongly influences the therapeutic effects of various drugs, compared to measurements in 2D cultures [3,9,10]. Notably, in spheroids with significantly smaller diameter is the zonal organization can only be partially observed or is absent, as there is no diffusion-limited shortage of nutrients or oxygen. Studies comparing gene expression profiles of spheroids and 2D cultures to resected tumors revealed different patterns in genes associated with cell survival, proliferation, differentiation, and therapy resistance, demonstrating that spheroids more closely resembled the characteristics of *in vivo* tumors [3,6]. These differences between 2D and 3D cultures were extended to metabolic studies using spheroids from the gastrointestinal tract, showing a higher ATP-linked respiration and non-aerobic ATP production. Interestingly, the metabolic difference between 2D and 3D cultures appeared to outweigh even the differences between different cell lines of the same entity [7].

Tumor cells including glioblastoma, exhibit alterations in their metabolism and mitochondrial dynamics, supporting their rapid proliferation, growth, survival, and spreading [11]. Rapid energy production, macromolecular biosynthesis, and an acidification of the tumor microenvironment, supporting tumor expansion are results of the metabolic switch [12]. Tumor cells frequently exhibit a reprogramming from oxidative phosphorylation to glycolysis, even in the presence of oxygen, known as the Warburg effect [12]. However, most solid tumors, including glioma depend mainly on cytosolic ATP produced from glycolysis, rather than ATP derived from mitochondria [11]. Thus, in 3D models it is of importance to apply valuable techniques to study cellular metabolism in an optimal manner. In most studies focusing on spheroid metabolism omics and tracing approaches were used, but studies on metabolic flux, in the form of oxygen consumption rates (OCR) and extracellular acidification rates (ECAR) are scarce [7]. OCR and ECAR provide valuable, time resolved, and thus easily combinable with interventions, measurements for the aerobic mitochondrial respiration and anaerobic glycolysis, respectively [1]. Yet, there are only very few studies establishing systematic protocols trying to optimize the procedure to measure metabolic flux rates in 3D cultures [13–17].

Here, we present a framework for performing metabolic measurements of 3D glioblastoma spheroids starting with the selection and optimization of the spheroid generation techniques for two standard cell lines and two primary cell lines, considering the reproducibility, the three-dimensional morphology and compatibility with transfer to the measurement device. In addition, we evaluated various normalization approaches to minimize difference between individual measurements and improve the signal-to-noise ratio.

## Methods

### Cell culture

The study was conducted in accordance with the Declaration of Helsinki and was approved by the local ethics committee of the University Halle-Wittenberg (project reference number: 2015−144, start: 04/12/2015-ongoing). All patients provided signed written informed consent. Primary cultures of brain tumor cells (designated as GBM #4, GBM #10) were obtained from human biopsies.

For experiments, LN229, U138, GBM#4 and GBM#10 glioblastoma cells were used. LN229 cells were purchased from the American Type Culture Collection (ATCC, CRL-261, Manassas, VA, USA), and U138 cells were obtained from Cell Lines Service (Cell Lines Service, 300363, Eppelheim, Germany). The primary glioblastoma lines #4 (GBM#4) and #10 (GBM#10) were isolated from human brain tumor biopsies, as described previously [18]. LN229 cells were cultured using 89% (v/v) Roswell Park Memorial Institute medium (Lonza, Basel, Switzerland, BE12-115F), supplemented with 10% (v/v) fetal bovine serum (FBS, Gibco, Carlsbad, CA, USA, 10500−064) and 1% (v/v) penicillin/streptomycin (P/S, Gibco, Carlsbad, CA, USA, 15140−122). U138, GBM#4 and GBM#10 were cultured in 89% (v/v) Dulbecco’s Modified Eagle Medium (Invitrogen, Waltham, MA, USA, 41965−062), and supplemented with 10% (v/v) FBS and 1% (v/v) P/S.

### 3D spheroid culture assays and analysis

Three-dimensional tumor aggregates were generated using three distinct methods; all based on the liquid overlay technique, and were performed in 96-well plates. A 4% (v/v) agarose was used to create a non-adhesive substrate in the first approach. The other two methods employed either the BioFloat system of Sarstedt (Sarstedt, Nümbrecht, Germany, 83.3925.400) or the 96-well bioprinting set of Greiner Bio One (Greiner, Kremsmünster, Austria, 655840). For bioprinting, 400.000 cells were seeded in a T25 culture flask. After 24 h, 80 µl of NanoShuttle, containing magnetic beads, was added to magnetize the cells overnight prior to spheroid generation. Throughout the figures, these techniques will be labeled as “Agarose”, “Bio Float” or “Magnetic Beads”, respectively.

To evaluate the quality of spheroid generation across the different methods 5,000, 10,000 and 20,000 cells were seeded onto the non-adhesive substrate. For the bioprinting assay, magnetized cells were placed on the magnetic drive for 1 h to initiate the spheroid formation. The cell aggregation process was monitored over a period of three days, with images captured every 30 min, using a Leica DMi8 with the LAS X control software (both: Leica, Wetzlar, Germany). Spheroid aggregation was analyzed as described previously [19,20].

In short, spheroids were segmented using a level set function based on the Chan-Vase energy [21] without any modification followed by morphological dilation, filling of holes and morphological erosion. Based on these segmentations spheroid size and circularity *c* was measured as $c=4*\pi *A/U^{2}$, for a spheroid of area *A* and circumference *U*. The segmentation and analysis procedure were implemented in Matlab 2021a and are available from the corresponding author on request. Additionally, we calculated the yield of spheroids per well for each cell type and condition, to assess the reliance of each technique, as the number of wells containing exactly one spheroid of circular shape divided by the total number of wells. Thus, the best possible yield would be one, corresponding to each well producing exactly one spheroid. Notably, all wells with multiple spheroids were excluded, as this does significantly affect the final size of the used spheroids.

Cell numbers of 20,000, 25,000 and 30,000 were selected for spheroid generation for the metabolic assay based on experimental and biological reasons. At these cell densities, the resulting spheroids were well visible, easily transferable, and large enough to develop different phenotypic, functional, and metabolic zones inside the spheroid.

To assess spheroids height, and thereby confirm their true three dimensionality, an atomic force microscope (AFM, Bruker, Billerica, MA, USA, Bioscope Catalyst) was employed. Spheroids were first transferred from 96-wells to petri dishes. For height measurements, a tipless cantilever (Arrow-TL2, Nanoworld, Neuchatel, Switzerland) was engaged to the spheroid, and the position of the stepper motor was measured. The cantilever was then engaged to the underlying substrate and the stepper motor height was measured again. The difference between these two values corresponds to the height of the spheroid.

### Spheroids metabolic analysis

After three days of aggregation, the spheroids were metabolically analyzed using Seahorse XF96 analyzer and the Mito Stress Test Kit (Agilent Technologies, Santa Clara, CA, United States). For the complete Seahorse assay medium, Seahorse XF RPMI or DMEM (pH 7.4) was supplemented with 10 mM D-glucose, 1 mM sodium pyruvate, and 2 mM L-glutamine.

The spheroids were transferred into Seahorse 96-well spheroid microplates (Agilent Technologies, Santa Clara, CA, United States) pre-coated with D-Poly-L-lysine at a concentration of 10 µg/ml (Thermo Fisher Scientific, Waltham, MA, United States). For the transfer, pipette tips were cut to gently guide the spheroids to the bottom of the wells. The spheroids were allowed to settle at the center of each well either by gravity and magnetic alignment or by gravity and gently tapping the pipette tip. Afterwards, each well was filled up to a total volume of 180 µl with assay medium.

Spheroid size, area, and position were determined using digital microscopy with the Cytation 1 Cell Imaging Multi-Mode Reader 1 operated via the Cell Imaging software (Agilent Technologies, Santa Clara, CA, United States).

For metabolic analysis, assay compounds were rehydrated in the assay medium to final concentrations of 2.5 µM oligomycin, 2.5 µM FCCP, and 0.8 µM rotenone/antimycin A.

The assay was performed using the Wave software. For the baseline measurement and following the addition of FCCP, five measurement cycles were conducted. After the addition of oligomycin and rotenone & antimycin A, eight cycles were performed for each.

For clustering of the Seahorse data, the following parameters were used as input for t-SNE analysis: spare capacity, maximal respiration, ATP-linked respiration, basal respiration, proton leak, OCR FCCP, OCR baseline, OCR oligomycin, and OCR rotenone.

### Normalizing seahorse data

To optimize the data analysis process, we employed three different cell numbers for spheroids of each cell type and applied multiple normalization strategies. For assessing the effect of the normalizations, 2D mono-layers of U138 and LN229 were used as reference. These cells were analyzed at a final concentration of 2 µM oligomycin, 1.5 µM FCCP, and 0.5 µM rotenone/antimycin A, following the manufacturer’s protocol. Cell numbers were quantified using Hoechst 33342 staining (6 µM/well end concentration; Thermo Fisher Scientific, Germany), and results were normalized accordingly using the digital microscope Cytation 1 Multi-Mode Reader and the software Wave together with the software Cell Imaging (Agilent Technologies, Santa Clara, CA, United States).

The reasoning for this approach was three-fold: 1) Using multiple cell numbers for spheroid formation should yield a cell number dependence of the basal and maximal respiration. 2) An effective normalization parameter should not only reduce variance between multiple cell numbers or spheroid sizes, but should also be applicable across different cell types. 3) Comparison with 2D measurements allowed estimating the currently available best possible case and an assessment of unavoidable biological and technical variance.

For normalization, data were first adjusted within each experiment and cell line. Therefore, the median of maximal respiration was calculated for each cell number within a given cell line. For each experiment, three median values per cell type were determined, corresponding to one per cell number used. Afterwards, the mean value of the three median values obtained for each cell line were used as a normalization factor to normalize the whole experiment for a given cell line. The way of normalization will be referred to as “median” in the figures or “median normalization” throughout the text.

After median normalization, all values were either kept unchanged, normalized to the cell number used for spheroid generation, or normalized to the area of the spheroid. To evaluate the effectiveness of each normalization method, the standard deviation relative to the mean value was used. Notably, a normalization was abandoned based on the surface area that could have been estimated using the measured spheroids heights. This is because only a fixed height value for each spheroid could be used, based on the respective cell line, e.g., Height = Diameter * Diameter_To_Height_Ratio. This method would not change the correlation or relative variance, compared to the normalization to the projected area measured above, as both follow the same scaling law, when using this approach for spheroid height estimation. Likewise, normalization to spheroid volume or cell number inside a spheroid, based on single cell volume will not yield different correlations or relative variances, for the same reason.

### Statistics

Statistics were performed in GraphPad Prism using a two-tailed ANOVA with the Bonferoni post-hoc test or the two-sided t-test. Significance was defined for p < 0.05. All error-bars and shaded areas depict the standard error of the mean. Experiments were repeated at least three independent times.

## Results

### Spheroid assay evaluation

**Fig 1:**
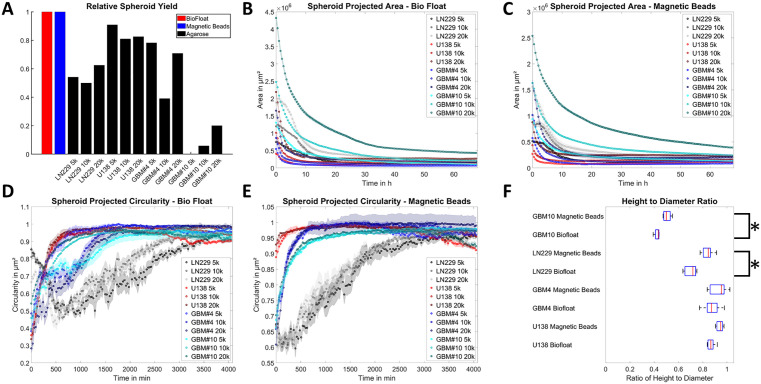
Spheroid Aggregation. A) Number of formed spheroids. Here, the number of single spheroids of roundish shape obtained per well for three tested methods used for the four glioblastoma cell lines and three different cell numbers is depicted. For BioFloat and magnetic bead technique all yields were equal to one and thus pooled. The 2D projected size is presented.(B, C). Graphs show the size of the formed spheroids as a function of time for the BioFloat (B) and magnetic bead (C) method. The circularity of the formed spheroids is shown as a function of time for the BioFloat (D) and magnetic bead (E) method. The relative height of spheroids for different techniques and all cell lines is presented (F). Error bars in B)-E) depict the standard error of the mean. Box plots in F) show the median (red line), the 25 and 75 percentile (blue box) and the maximal range (whiskers). Stars depict statistically significant results with p < 0.05. B) to E) For each cell line and cell number 16 spheroids were analyzed. F) n_GBM10 Beads_ = 8, n_GBM10 Biofloat_ = 8, n_GBM4 Beads_ = 12, n_GBM4 Biofloat_ = 9, n_LN229 Beads_ = 11, n_LN229 Biofloat_ = 8, n_U138 Beads_ = 12, n_U138 Biofloat_ = 8.

First, we measured how many spheroids were obtained per well seeded for each technique, cell type and seeded cell number. There, we could demonstrate that the yield was by far lowest when using the agarose method for forming a non-adhesive surface. Depending on the cell line, less than 25% (GBM#10) and up to approximately 80% (U138) of all wells formed usable spheroids, meaning that exactly one spheroid of roundish shape was formed per well. For the agarose technique multiple spheroids per well were generated often, leading to spheroids of varying size (S10 Fig), potentially affecting metabolic read-outs, as the nutrient and oxygen-supply of cells inside the spheroid is size-dependent [3,9]. In contrast, the two other methods, namely BioFloat and Magnetic Beads, achieved a consistent yield of 100% for each condition and cell line (Fig 1A). Consequently, further analyses were carried out for the BioFloat and Magnetic Beads technique only.

Analyzing the aggregation dynamics of the spheroids, both remaining techniques were forming spheroids within a comparable period. Regarding the initial sizes, BioFloat spheroids were significantly larger for LN229, U138 and GBM#10 but not for GBM#4. This is most likely due to initial magnetically induced aggregation when using magnetic beads within the bio-printing technique (Fig 1B-C). Interestingly, the size difference was not maintained for the equilibrium sizes, when the spheroids were neither significantly growing nor shrinking anymore. The spheroid size was comparable for spheroids from both methods with differences mostly ≤15%, roughly corresponding to the cell counting differences expected when using a Countess system for cell number estimation, as was done here (Fig 1B-C, S1 Table) [22]. Analyzing the shape, the bio-printed spheroids reached significantly faster a round equilibrium state than the BioFloat spheroids, albeit both formed almost perfectly round spheroids for all cell lines at the end of the measurement period (Fig 1D-E). Notably, LN229 and GBM#10 spheroids appeared less compact than those of U138 and GBM#4, as individual cells were still visible at the border of the spheroids (S1 Fig). To characterize the three-dimensional structure of the formed spheroids the height to diameter ratio was measured using an AFM (atomic force microscope). The height to diameter ratio was cell line dependent and found to range from approximately 0.5 (GBM#10) and 0.8 (LN229) to 0.95 (U138 and GBM#4), respectively. This observation also agrees with the formation of less compact spheroids of LN229 and GBM#10. Notably, for all cell lines the spheroids formed using magnetic beads were more spherical, with higher height to diameter ratios. This was especially noteworthy and statistically significant for LN229 and GBM#10 (*p < 0.05, Fig 1F).

The bio-printing and Bio Float technique were therefore considered to form usable and reproducible spheroids suitable for further measurements.

### Positioning of spheroids in measurement plates

Before performing metabolic measurements with spheroids generated by both techniques, the accuracy of the spheroid position was assessed inside the measurement plates necessary for performing the metabolic analysis. For the BioFloat system, spheroids were placed manually via pipetting and moving into the wells center spheroids into the measurement plate and subsequently. As the bio-printed spheroids were magnetic, they were pipetted into the measurement plate, which was placed onto the magnetic drive used for initial aggregation, to center the spheroids. Counting the number of spheroids in or close to the center of the measurement well (S2 Fig), we found that the bio-printed spheroids were more centered, with 86% (238/276) found in or near the center, compared to 52% (48/92) for the Bio Float method. Using the magnetic spheroids, the transfer process was faster and more reproducible, as their position remained stable during handling on the magnetic drive. Thus, subsequent measurements were carried out using the bio-printed spheroids.

### Seahorse measurement results

**Fig 2:**
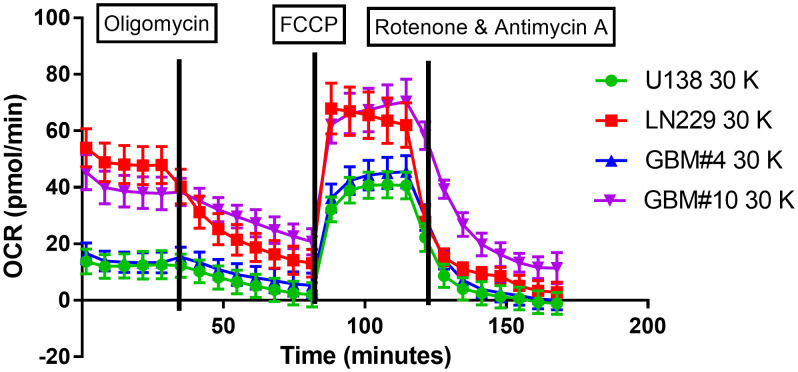
Seahorse analyses. Seahorse analyses showing a comparison between metabolic values in the different glioblastoma cell lines LN229, U138 and the primary cells GBM#4, GBM#10. Representative mitochondrial respiration measurement using the Seahorse XF Cell Mito Stress Test for U138, LN229, GBM#4 and GBM#10 spheroids generated from 30,000 cells. Oligomycin, FCCP, rotenone plus antimycin A are used as pharmacological tools to study mitochondrial respiration. Oligomycin inhibits ATP synthase, reducing oxygen consumption. Rotenone and antimycin A block the electron transport chain, preventing mitochondrial respiration and allowing measurement of non-mitochondrial oxygen consumption. Oligomycin reduces the oxygen consumption rate (OCR) through inhibition of ATP synthase. FCCP uncouples the proton gradient, maximizing oxygen consumption. Rotenone and antimycin A are electron transport chain inhibitors.

**Fig 3:**
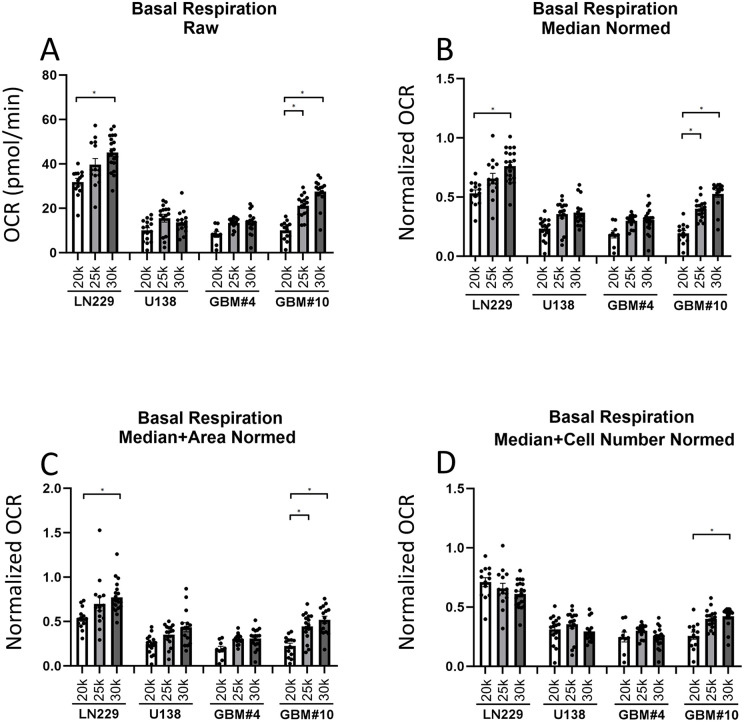
Basal Respiration. (A) Graphic of the raw basal respiration data obtained from the Seahorse metabolic analysis. The data were normalized using different approaches and are shown in (B) for median normalization, (C) for median plus area normalization, and (D) for median plus cell number normalization. Error bars represent the standard error of the mean (SEM). Asterisks indicate statistically significant differences at p < 0.05. The number of samples used in each group was as follows: n_GBM10 15k_ = 13, n_GBM10 20k_ = 16, n_GBM10 25k_ = 13, n_GBM4 15k_ = 12, n_GBM4 20k_ = 15, n_GBM4 25k_ = 17, n_LN229 15k_ = 13, n_LN229 20k_ = 14, n_LN229 25k_ = 20, n_U138 15k_ = 16, n_U138 20k_ = 15, n_U138 25k_ = 17.

**Fig 4:**
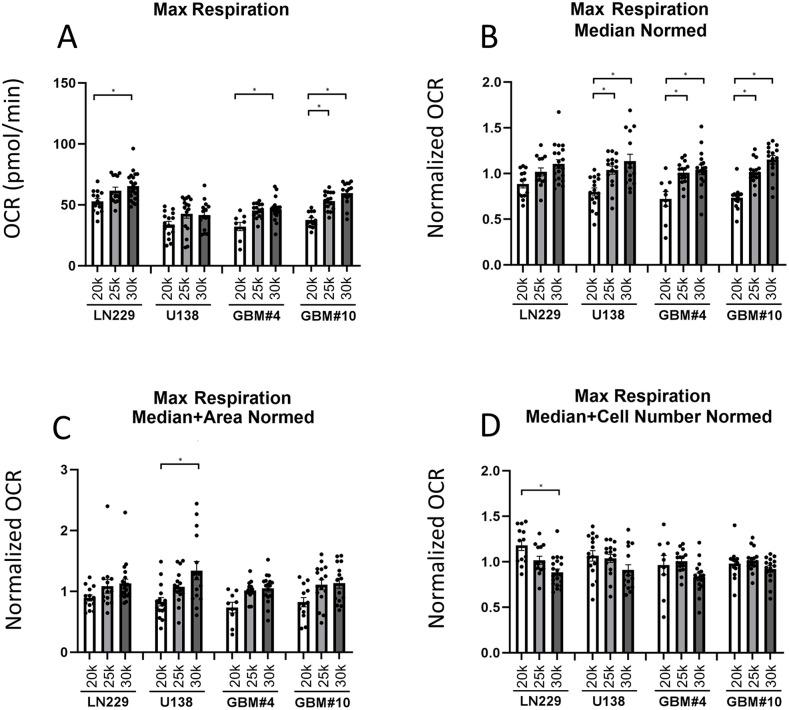
Maximal Respiration. Seahorse analyses showing the differences in the maximal respiration values of glioblastoma cell lines LN229, U138 and the primary cells GBM#4, GBM#10 after normalization of spheroids yielded from different cell seeding densities. (A) Graphic of the raw maximal respiration data obtained from the Seahorse metabolic analysis. The data were normalized using different approaches and are shown in (B) for median normalization, (C) for median plus area normalization, and (D) for median plus cell number normalization. Error bars represent the standard error of the mean (SEM). Asterisks indicate statistically significant differences at p < 0.05. The number of samples used in each group was as follows: n_GBM10 15k_ = 13, n_GBM10 20k_ = 16, n_GBM10 25k_ = 13, n_GBM4 15k_ = 12, n_GBM4 20k_ = 15, n_GBM4 25k_ = 17, n_LN229 15k_ = 13, n_LN229 20k_ = 14, n_LN229 25k_ = 20, n_U138 15k_ = 16, n_U138 20k_ = 15, n_U138 25k_ = 17.

**Fig 5:**
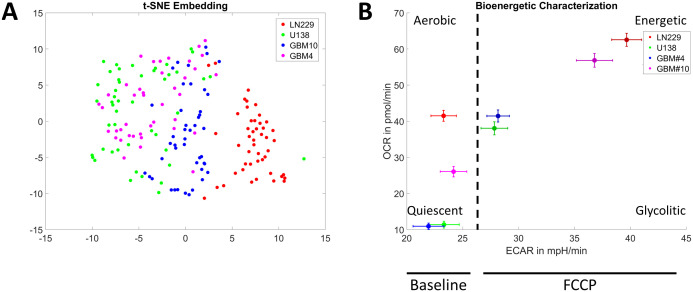
Clustering of cell types. (A) t-SNE clustering of spheroids from the four cell lines, showing the distinct response of LN229 compared to the other GBM cell types. Each point represents a single spheroid. Each point corresponds to the data of a single spheroid. (B) Illustration of the energetic state of the four GBM spheroid types at baseline and after FCCP injection to achieve maximal respiration. Error bars represent the standard error of the mean (SEM). The following number of spheroids was used for each group: n_GBM10k_ = 42, n_GBM4_ = 44, n_LN229_ = 47, n_U138_ = 48.

To investigate differences in *in vitro* metabolism between various cell lines in 3D culture and across different cell numbers, the Seahorse XFe96 analyzer was used to measure the oxygen consumption rate (OCR) of each cell line. The metabolic profiles of established GBM tumor cell lines (LN229 and U138) were compared to those of primary cells (GBM#4 and GBM#10), dependent on the spheroid size (Figs 2–5, S3–S8).

To confirm the reliability of the metabolic profiles, spheroids were treated with established modulators of mitochondrial function—oligomycin, FCCP, and rotenone/antimycin A, observing the expected shifts indicating a fully functional system (Figs 3, 4). The four cell lines differed in their metabolic behaviors depending on the cell line and cell numbers.

The metabolic analysis of different spheroids showed for the cell line LN229 a higher basal respiration rate compared to other cell lines, indicating an elevated baseline metabolic activity. While the maximal respiratory capacity was similar across all cell lines, the spare respiratory capacity was reduced in the LN229 cell line, suggesting a limited ability to respond to increased energy demands (Figs 3, 4). Additionally, ATP production was elevated, whereas coupling efficiency remained comparable to the other cell lines (S3–S9 Figs).

These findings suggest that the LN229 spheroids operate at a higher metabolic level under basal conditions and produce more ATP, but at the cost of reduced metabolic flexibility. The diminished spare respiratory capacity may hint to a higher susceptibility to energetic stress, as the cells have less reserve to adapt to increased energy requirements.

For further characterization of the metabolic landscape of the spheroids from different cell lines, all direct and indirect OCR-related read-outs of the Seahorse measurements were used to cluster the spheroids using t-distributed Stochastic Neighbor Embedding (t-SNE). LN229 were distinct of other cell lines, and U138 and GBM#4 clustered roughly together. In contrast, GBM#10 appeared to take an intermediate spot between LN229 and GBM#4/ U138. Thus, the four used cell lines reflect the heterogeneity of GBM, showing a broad, cell line dependent spectrum of metabolic responses in the used assay (Fig 5A). Further bio-energetic characterization of the four spheroids types in basal conditions revealed, that U138 and GBM#4 were initially in a comparably quiescent state, while LN229 and GBM#10 were in a state of higher aerobic respiration. Following FCCP treatment, a mitochondrial uncoupler that simulates physiological energy demand by stimulating maximal respiratory capacity, spheroids from all four different cell lines displayed an elevated metabolic state in both oxidative phosphorylation and glycolysis. This effect was particularly pronounced in LN229 and GBM#10 spheroids (Fig 5B).

Analyzing the basal respiration, a significant increase with cell number was observed for LN229 and GBM#10 across all normalization approaches. However, a similar trend was not seen for U138 and GBM#4 between 20.000 and 25.000 cells, indicating that the test had reached a saturation level, potentially due to spheroids reaching a critical size, in which additional cells or spheroid size would largely just yield to an increase of the quiescent cell population (Fig 3). These results suggest that, for each cell line, optimizing the appropriate cell count is essential before starting the seahorse assay. The results for baseline, FCCP, rotenone, oligomycin, ATP measurements, coupling efficiency and spare respiratory capacity were similar (S3–S8 Figs).

### Normalization

**Fig 6:**
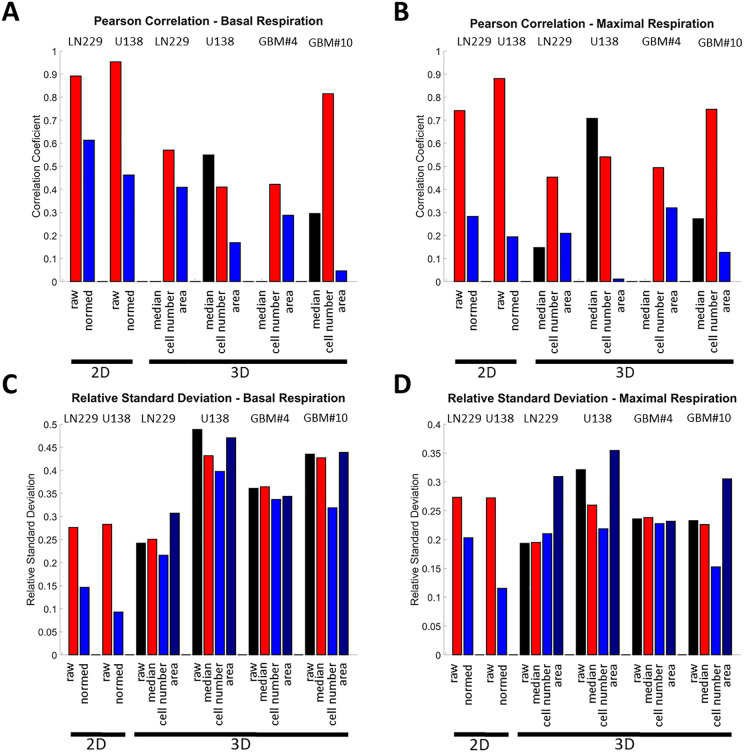
Results of different normalization approaches and comparison to 2D. (A) and (B) are graphs of the Pearson correlation coefficient of the raw or normalized OCR of basal (A) or maximal respiration (B) values with the cell number for 2D and 3D spheroids measurements. C) and D) are graphs of the relative variance of the raw or normalized OCR of basal (A) or maximal respiration (B) values for the 2D and 3D spheroids measurements.

Since systematic inter-experimental variance, caused by, e.g., slight variations in the cell density estimation due to cell counting, leading to different numbers used to generate spheroids, and others occurs, the data analysis was optimized comparing four different normalization approaches. First, Pearson correlation coefficients were calculated between the raw data and all other normalization approaches. For comparison, for the 2D assays, the raw data was correlated with the calculated cell number from the DAPI staining and the normalized data was correlated with the cell numbers, to evaluate if complete de-correlation was possible. The correlation analysis demonstrated that both the basal and maximal respiration of the 2D assays was, as expected strongly correlated (r ≥ 0.75) with the underlying cell number. Yet, normalization could only partially de-correlate the data from the total cell number, with residual correlation coefficients of 0.2–0.3 for maximal respiration and 0.45–0.6 for basal respiration (Fig 6A-B).

For the normalization of the 3D measurements, the normalization approaches showed varying degrees of correlation. Correlation coefficients for normalization to cell numbers ranged from 0.4 to 0.8, showing highest correlation coefficients. For the other approaches, correlation coefficients showed either low or strongly variable correlation (Fig 6A-B).

Next, the normalized standard deviation was analyzed. For 2D measurements, normalization to cell number reduced the relative standard deviation from approximately 0.25–0.3 to around 0.1–0.2 demonstrating the method#39;s reliability (Fig 6C-D). For the 3D spheroids, initial variation ranged from about 0.2 to 0.48, without normalization but it was reduced to a range of about 0.15 to 0.4 with the normalization to cell number. Again, the same pattern as for the correlation analysis was visible, with the normalization to the combination of median and subsequent cell number being most effective (Fig 6C-D). Noteworthy, even though the variance is apparently higher than for 2D measurements, the normalization approaches led to a reduction of the variation, yielding variations that are comparable to 2D measurements for the maximal respiration. Of note, the basal respiration values for U138 spheroids should be interpreted with caution, as they were often close to the lower detection threshold, and therefore were likely more error-prone.

## Discussion

In this study, we optimized the methodological procedure for analyzing 3D spheroids using the Seahorse XF96 device to characterize metabolic activity and treatment response of glioblastoma cells. Different methods for generating 3D cultures were evaluated and compared to determine the most effective approach in terms of yield and reproducibility. In addition, the efficiency for spheroid transfer was assessed across different generation methods. Finally, an optimization of the data analysis pipeline was achieved with focus on normalization of the metabolic measurements.

When measuring metabolic activity of 2D cultures with a height of approximately 10 µm, all cells were equally covered and emerged in the respective measurement medium. Under these conditions a limited diffusion of nutrients, treatment substances etc. can be neglected, making it feasible to normalize measurement values to the total cell number analyzed [1]. Thus, for such an analysis it is implicitly assumed, that all analyzed cells behave the same. Yet, the same approach cannot be easily transferred to 3D cultures, for multiple reasons. Culturing cells in 3D, compared to standard cell culture plates, leads to cells having on average a higher amount of cell-cell adhesions, lower cell-matrix adhesion and experiencing a more compliant environment [23].

Thus, phenotypic changes and quantitative or even qualitative differences compared to 2D cultures are very likely [7]. Therefore, direct comparison to 2D cultures can be lackluster. In sufficiently large and complex 3D aggregates, central regions may even experience nutrient and oxygen deprivation, dismissing the implicit assumption of all cells inside a 3D aggregate behaving identically [3,9]. Furthermore, patient-derived GBM organoids showed to preserve *in vivo*-like metabolic programs, including enhanced mitochondrial metabolism and metabolic plasticity, which are largely lost in standard 2D culture systems [24]. These findings support the concept that glycolytic and oxidative metabolic phenotypes coexist within GBM tumors and are more accurately recapitulated in 3D culture models than in 2D monolayers [25]. GBM spheroids show metabolic heterogeneity with glycolytic shifts under microenvironmental stress, more representative of *in vivo* conditions [26].

Consequently, normalization of measurement values for 3D aggregates to the cell number is questionable. Interestingly, this also implies that it is necessary to assess the actual 3D structure of spheroids, as the 2D circumference of a 3D spheroid is not necessarily a good estimate for its actual height – as shown in this study – and thus for the amount of diffusion limited supply for cells in the core of the aggregate. Previous studies analyzing different methods to normalize Seahorse data for 3D spheroids have predominantly relied on 2D imaging data [13–15,27] without accounting for true spheroid height. This limitation is particularly relevant for studies employing relatively low cell numbers, resulting in spheroids of large diameter, implying a low height and thus little to no diffusion limitations of nutrients [13,15,17,27].

Additionally, in previous studies, normalization to essentially 2D parameters agnostic to actual spheroid height yielded best normalization results [13,14,17,27], giving rise to the speculation that this effect is actually due to cells in the aggregate not being limited by diffusion due to insufficient aggregate height. In contrast, in our study, the best normalization was not achieved when using the projected spheroid area, but rather by normalizing to the initial cell number. Notably, the height of the used spheroids in this study varied between 300 µm (GBM#10) and 530 µm (U138), demonstrating true three dimensionality. Interestingly, the normalization to cell number had the largest effect on GBM#10, having the lowest diameter, supporting the previous argument that diffusion limitations are less pronounced in smaller aggregates. Another source of inaccuracy in this approach may stem from the proliferation occurring between cell seeding and collection of spheroids. However, our previous study demonstrated only very few proliferating cells in tumor spheroids of comparable size (≤5%), making this a minor source of error [19]. This also is in line with our observation here that spheroids reached, depending on the cell line, a stable size, without significant growth, after approximately 20–40 h. Of note, while the normalization to the initially seeded cell number worked best in this study, it did have a lower efficiency in lowering variance, compared to the established procedure in 2D cultures, used as a gold standard. Therefore, we assume that another optimal normalization factor exist that should theoretically bring the variation approximately in line with those of the 2D measurements. We suspect that the compactness of the spheroid, as well as its three-dimensional shape and size play main roles in it, as those limit diffusion.

Our results demonstrated that for the metabolic analysis of 3D spheroids, it is essential to generate aggregates with uniform diameters in order to obtain reproducible results. The use of magnetic beads was shown to offer several advantages. This technique enables the formation of spheroids with a defined cell number, allowing for highly reproducible measurements with low standard deviations — while considering that each cell line forms spheroids with its own specific characteristics.

Moreover, for analyzing the metabolic behavior of cells using the Seahorse device, spheroids must be transferred to the measurement plate. Thanks to the magnetic system, it was possible to reliably center the spheroids within the wells. As a result, the proportion of spheroids suitable for measurements was significantly higher compared to the other evaluated method.

The use of Seahorse XF Analyzers to assess cellular metabolism in 3D spheroids is a promising approach. The measurement of OCR provides valuable information about mitochondrial metabolism. Mito Stress Assay is a suitable method for characterizing the mitochondrial function of spheroids, which can vary between different cell lines. This approach is particularly useful for comparing various cell lines, as well as for assessing the effects of different drugs or inhibitors on the spheroids.

On the other hand, it comes with important methodological limitations. Reliable quantification of ATP production from both glycolysis and mitochondrial oxidative phosphorylation requires accurate determination of the ECAR and specifically the partitioning of ECAR to CO₂-derived acidification and glycolytic acidification. This is typically achieved by calculating the proton efflux rate (PER), which depends on validated buffer capacity factors for the specific plate and assay setup. While ECAR values measured during the Mito Stress Test may provide a general indication or trend of glycolytic activity, it cannot be used to accurately quantify glycolytic ATP production. Without proper correction for CO₂-derived acidification and without validated buffer capacity parameters, ECAR measurements only reflect overall extracellular acidification and therefore can indicate the direction or tendency of glycolytic engagement rather than providing a precise measure of glycolytic flux or ATP generation.

Furthermore, while the measurement of OCR provides valuable information about mitochondrial metabolism, it alone is insufficient to determine whether cells are exhibiting Warburg type metabolic reprogramming. By definition, the Warburg effect involves elevated glycolytic activity under aerobic conditions. Therefore, without the ability to quantify glycolytic rates through validated ECAR and PER measurements and to compare ATP production from both pathways, conclusions about the preservation of the Warburg effect in 3D spheroids remain incomplete.

## Conclusion and outlook

In conclusion, the Seahorse analyzer is a powerful tool for investigating cellular metabolism, in 3D spheroid cultures. In this study cell type and spheroid, size dependent responses to metabolic challenges in four glioblastoma cell lines were assessed. Using bio-printed magnetic beads based spheroids, combined with normalization to the initially seeded cell number, and median achieved the most reliable normalization results. This method provides straightforward and reproducible approach for generating of 3D cell cultures and optimizing metabolic measurements within these cultures.

## Supporting information

S1 Fig Sample images of spheroid aggregation. The images show typical spheroid aggregation, together with the detected edges, for all four cell lines at time points 0 h und 70 h, for 10,000 cells using the BioFloat technique. Please denote the visible individual cells at the edge of GBM#10 and LN229 spheroids after 70 h. The scale bar corresponds to 250 µm.
(TIF)

S2 Fig Scheme for assigning good positioning for Seahorse measurements. Sample images of the Seahorse well plates with the encircled green area showing the optimal spheroid position (top left). Spheroids inside this area (top right) or touching this area (bottom left), were considered to be centered sufficiently. Measurements of spheroids not in contact with the central structure were discarded (bottom right).
(TIF)

S3 Fig The comparison between different normalization methods for basal respiration. Basal respiration compared between different glioblastoma cell lines LN229, U138 and primary cells GBM#4, GBM#10 after normalization for 20,000, 25,000 and 30,000 cells. The raw values (A), median normed (B), median plus area normed (C), and median plus cell number normed (D) normalization approaches were used. Error bars correspond to the standard error of the mean. Stars depict statistically significant results with p < 0.05. The number of samples used in each group was as follows: n_GBM10 15k_ = 13, n_GBM10 20k_ = 16, n_GBM10 25k_ = 13, n_GBM4 15k_ = 12, n_GBM4 20k_ = 15, n_GBM4 25k_ = 17, n_LN229 15k_ = 13, n_LN229 20k_ = 14, n_LN229 25k_ = 20, n_U138 15k_ = 16, n_U138 20k_ = 15, n_U138 25k_ = 17.
(TIF)

S4 Fig The comparison between different normalization methods for ATP production. ATP production compared between different glioblastoma cell lines LN229, U138 and primary cells GBM#4, GBM#10 after normalization for 20,000, 25,000 and 30,000 cells. The raw values (A), median normed (B), median plus area normed (C), and median plus cell number normed (D) normalization approaches were used. Error bars correspond to the standard error of the mean. Stars depict statistically significant results with p < 0.05. The number of samples used in each group was as follows: n_GBM10 15k_ = 13, n_GBM10 20k_ = 16, n_GBM10 25k_ = 13, n_GBM4 15k_ = 12, n_GBM4 20k_ = 15, n_GBM4 25k_ = 17, n_LN229 15k_ = 13, n_LN229 20k_ = 14, n_LN229 25k_ = 20, n_U138 15k_ = 16, n_U138 20k_ = 15, n_U138 25k_ = 17.
(TIF)

S5 Fig The comparison between different normalization methods for maximal respiration. Maximal respiration compared between different glioblastoma cell lines LN229, U138 and primary cells GBM#4, GBM#10 after normalization for 20,000, 25,000 and 30,000 cells. The raw values (A), median normed (B), median plus area normed (C), and median plus cell number normed (D) normalization approaches were used. Error bars correspond to the standard error of the mean. Stars depict statistically significant results with p < 0.05. The number of samples used in each group was as follows: n_GBM10 15k_ = 13, n_GBM10 20k_ = 16, n_GBM10 25k_ = 13, n_GBM4 15k_ = 12, n_GBM4 20k_ = 15, n_GBM4 25k_ = 17, n_LN229 15k_ = 13, n_LN229 20k_ = 14, n_LN229 25k_ = 20, n_U138 15k_ = 16, n_U138 20k_ = 15, n_U138 25k_ = 17.
(TIF)

S6 Fig The comparison between different normalization methods for the oligomycin treatment. Oligomycin treatment compared between different glioblastoma cell lines LN229, U138 and primary cells GBM#4, GBM#10 after normalization for 20,000, 25,000 and 30,000 cells. The raw values (A), median normed (B), median plus area normed (C), and median plus cell number normed (D) normalization approaches were used. Error bars correspond to the standard error of the mean. Stars depict statistically significant results with p < 0.05. The number of samples used in each group was as follows: n_GBM10 15k_ = 13, n_GBM10 20k_ = 16, n_GBM10 25k_ = 13, n_GBM4 15k_ = 12, n_GBM4 20k_ = 15, n_GBM4 25k_ = 17, n_LN229 15k_ = 13, n_LN229 20k_ = 14, n_LN229 25k_ = 20, n_U138 15k_ = 16, n_U138 20k_ = 15, n_U138 25k_ = 17.
(TIF)

S7 Fig The comparison between different normalization methods for the different single parameters obtained from metabolic Seahorse analysis. Rotenone plus antimycin A treatment compared between different glioblastoma cell lines LN229, U138 and primary cells GBM#4, GBM#10 after normalization for 20,000, 25,000 and 30,000 cells. The raw values (A), median normed (B), median plus area normed (C), and median plus cell number normed (D) normalization approaches were used. Error bars correspond to the standard error of the mean. Stars depict statistically significant results with p < 0.05. The number of samples used in each group was as follows: n_GBM10 15k_ = 13, n_GBM10 20k_ = 16, n_GBM10 25k_ = 13, n_GBM4 15k_ = 12, n_GBM4 20k_ = 15, n_GBM4 25k_ = 17, n_LN229 15k_ = 13, n_LN229 20k_ = 14, n_LN229 25k_ = 20, n_U138 15k_ = 16, n_U138 20k_ = 15, n_U138 25k_ = 17.
(TIF)

S8 Fig The comparison between different normalization methods for coupling efficiency and spare respiratory capacity. The raw values (A, B) of coupling efficiency and spare respiratory capacity compared between different glioblastoma cell lines LN229, U138 and primary cells GBM#4, GBM#10 after normalization for 20,000, 25,000 and 30,000 cells. The number of samples used in each group was as follows: n_GBM10 15k_ = 13, n_GBM10 20k_ = 16, n_GBM10 25k_ = 13, n_GBM4 15k_ = 12, n_GBM4 20k_ = 15, n_GBM4 25k_ = 17, n_LN229 15k_ = 13, n_LN229 20k_ = 14, n_LN229 25k_ = 20, n_U138 15k_ = 16, n_U138 20k_ = 15, n_U138 25k_ = 17.
(TIF)

S9 Fig The comparison between different normalization methods for the different single parameters obtained from metabolic Seahorse analysis between all cell lines for 30,000 Spheroids. (A) Basal Respiration, (B) Maximal Respiration, (C) ATP, (D) FCCP, (E) Baseline and (F) Rotenone are shown. Error bars correspond to the standard error of the mean. The number of samples used in each group was as follows: n_GBM10 15k_ = 13, n_GBM10 20k_ = 16, n_GBM10 25k_ = 13, n_GBM4 15k_ = 12, n_GBM4 20k_ = 15, n_GBM4 25k_ = 17, n_LN229 15k_ = 13, n_LN229 20k_ = 14, n_LN229 25k_ = 20, n_U138 15k_ = 16, n_U138 20k_ = 15, n_U138 25k_ = 17.
(TIF)

S10 Fig The comparison between agarose and Biofloat technique. Example of LN229 spheroids generated with the agarose (left) and BioFloat (right) technique. Please denote the generation of multiple spheroids of different size with the agarose technique. Notably, all of those spheroids are significantly smaller compared to the case that all seeded cells formed exactly one spheroid (right image).
(PNG)

S1 Table Descriptive statistics. (DOCX)
