# Supplementary figures and images for: An optimized protocol for metabolic measurement in 3D tumor spheroids derived from primary and established glioblastoma cells
Source: PLoS One. 2026 Apr 24;21(4):e0347569. doi: 10.1371/journal.pone.0347569 (PMC13108750; doi:10.1371/journal.pone.0347569)

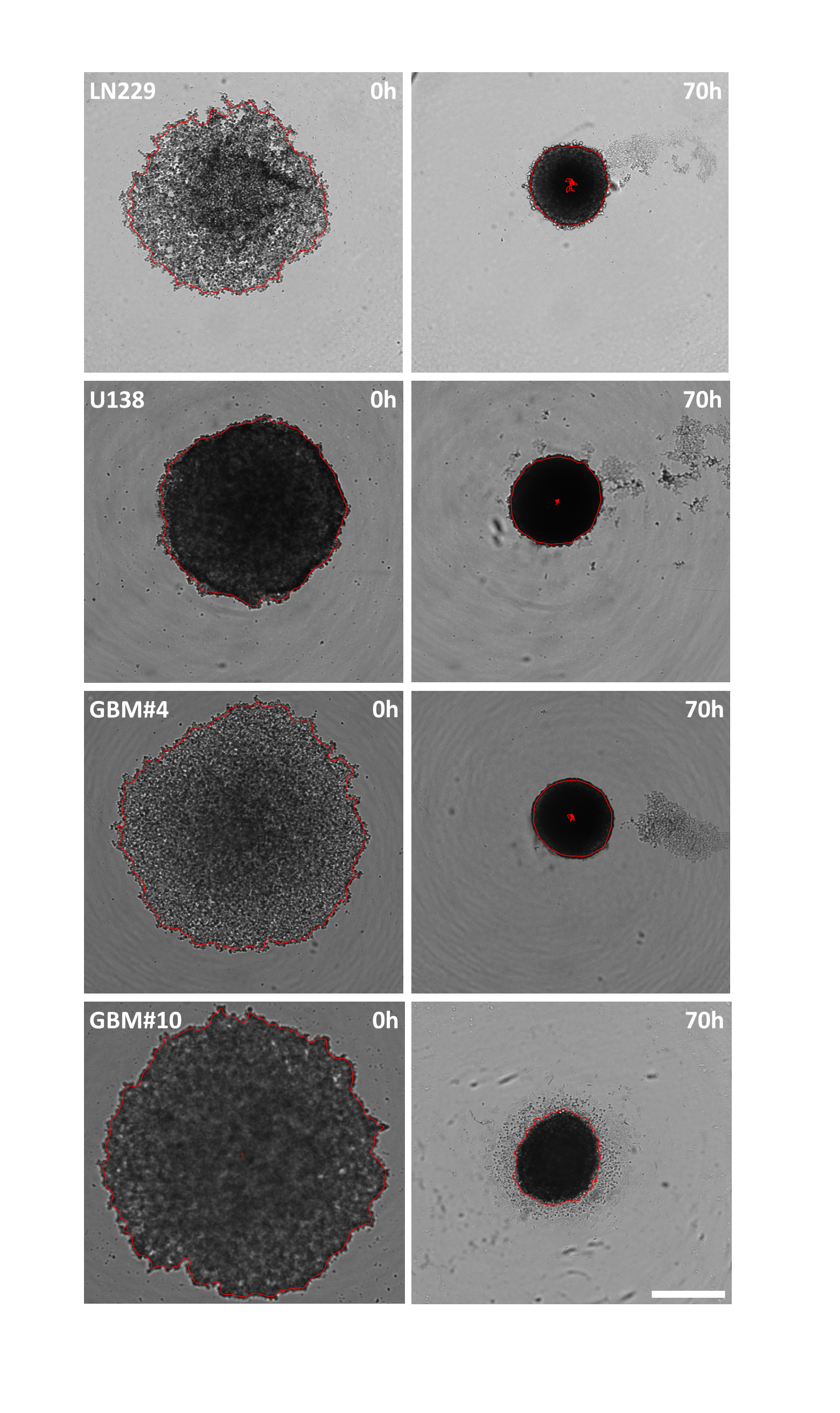

Supplement: S1 Fig — The images show typical spheroid aggregation, together with the detected edges, for all four cell lines at time points 0 h und 70 h, for 10,000 cells using the BioFloat technique. Please denote the visible individual cells at the edge of GBM#10 and LN229 spheroids after 70 h. The scale bar corresponds to 250 µm. (TIF) [file pone.0347569.s001.TIF]

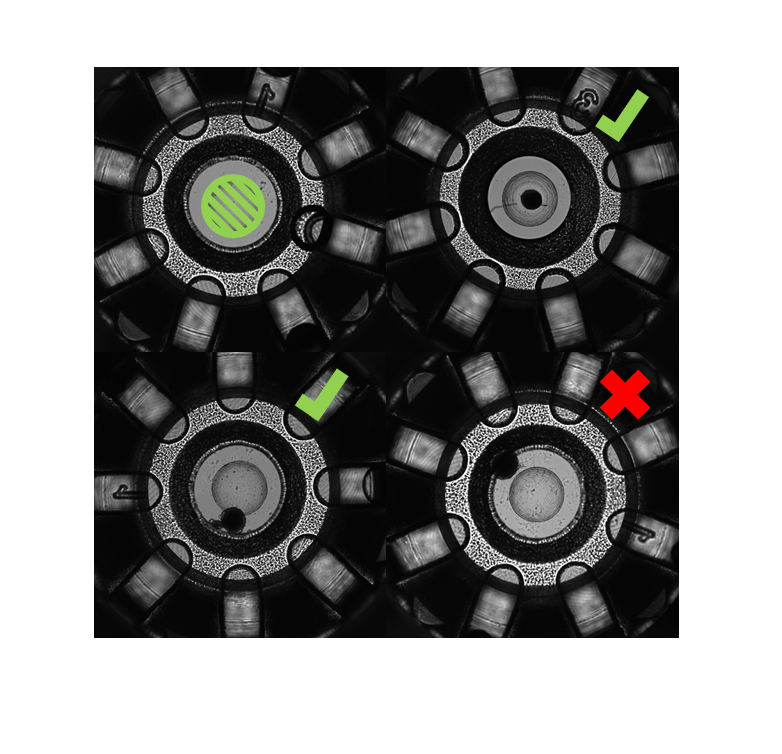

Supplement: S2 Fig — Sample images of the Seahorse well plates with the encircled green area showing the optimal spheroid position (top left). Spheroids inside this area (top right) or touching this area (bottom left), were considered to be centered sufficiently. Measurements of spheroids not in contact with the central structure were discarded (bottom right). (TIF) [file pone.0347569.s002.TIF]

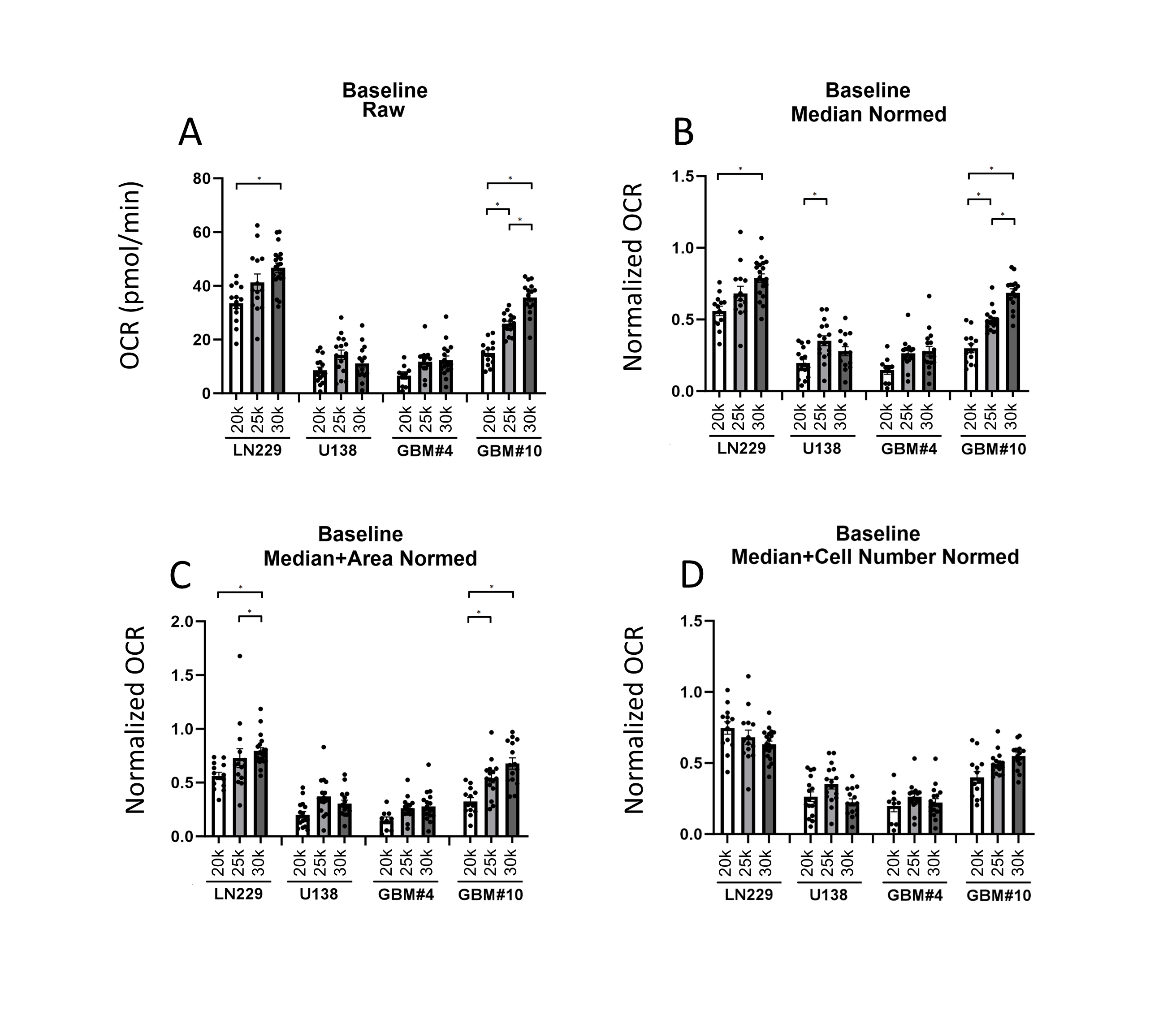

Supplement: S3 Fig — Basal respiration compared between different glioblastoma cell lines LN229, U138 and primary cells GBM#4, GBM#10 after normalization for 20,000, 25,000 and 30,000 cells. The raw values (A), median normed (B), median plus area normed (C), and median plus cell number normed (D) normalization approaches were used. Error bars correspond to the standard error of the mean. Stars depict statistically significant results with p < 0.05. The number of samples used in each group was as follows: nGBM10 15k = 13, nGBM10 20k = 16, nGBM10 25k = 13, nGBM4 15k = 12, nGBM4 20k = 15, nGBM4 25k = 17, nLN229 15k = 13, nLN229 20k = 14, nLN229 25k = 20, nU138 15k = 16, nU138 20k = 15, nU138 25k = 17. (TIF) [file pone.0347569.s003.TIF]

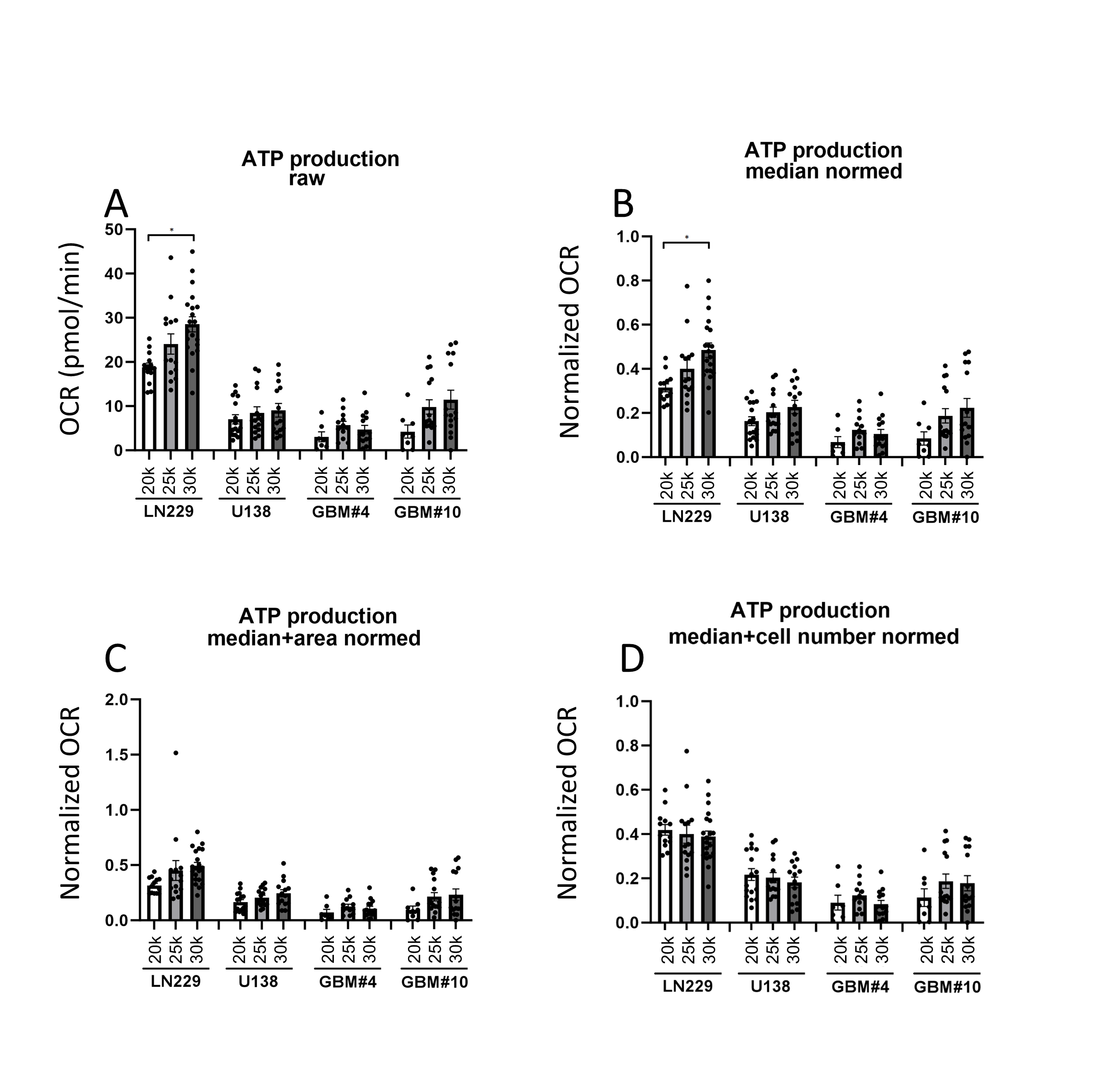

Supplement: S4 Fig — ATP production compared between different glioblastoma cell lines LN229, U138 and primary cells GBM#4, GBM#10 after normalization for 20,000, 25,000 and 30,000 cells. The raw values (A), median normed (B), median plus area normed (C), and median plus cell number normed (D) normalization approaches were used. Error bars correspond to the standard error of the mean. Stars depict statistically significant results with p < 0.05. The number of samples used in each group was as follows: nGBM10 15k = 13, nGBM10 20k = 16, nGBM10 25k = 13, nGBM4 15k = 12, nGBM4 20k = 15, nGBM4 25k = 17, nLN229 15k = 13, nLN229 20k = 14, nLN229 25k = 20, nU138 15k = 16, nU138 20k = 15, nU138 25k = 17. (TIF) [file pone.0347569.s004.TIF]

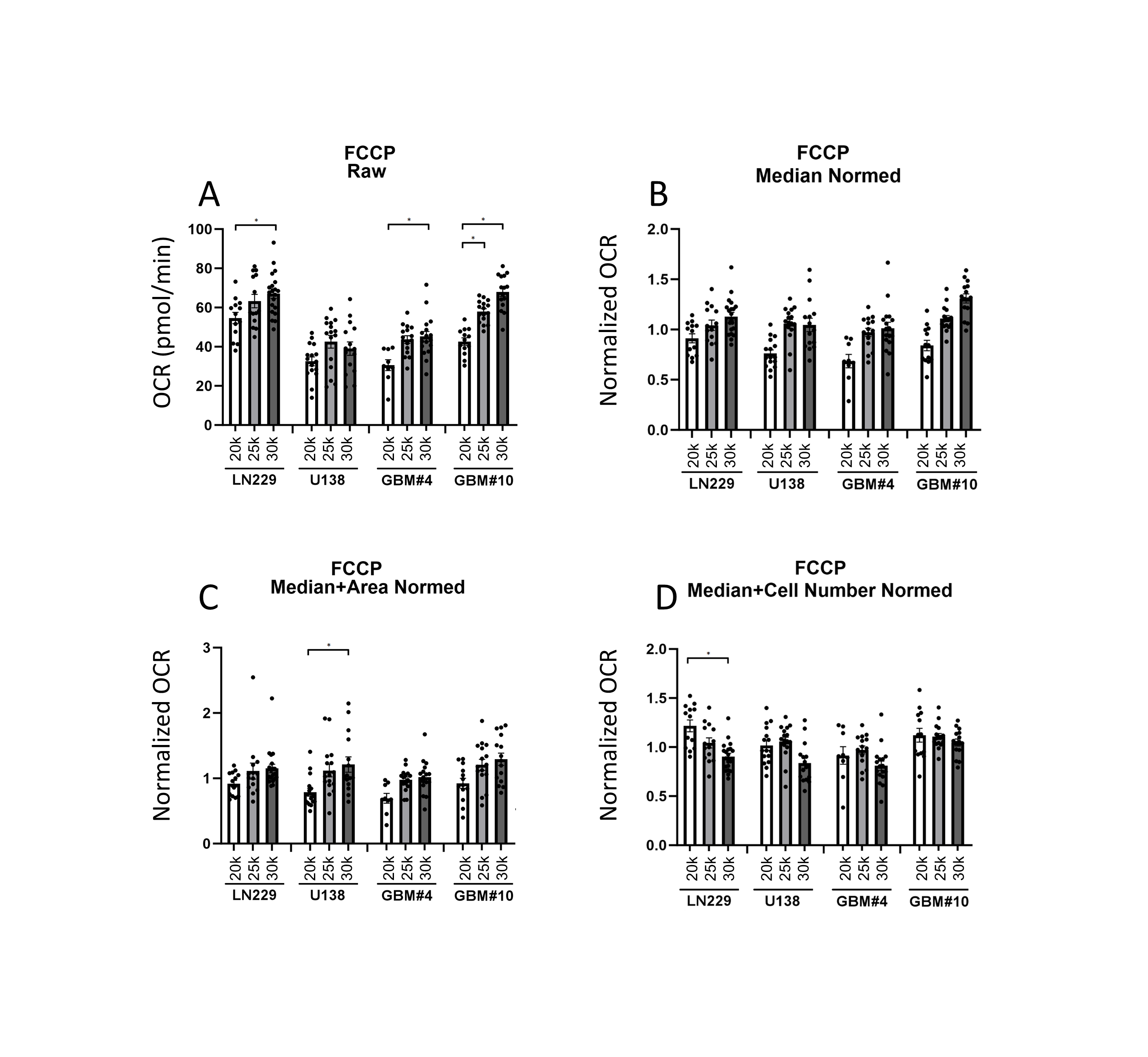

Supplement: S5 Fig — Maximal respiration compared between different glioblastoma cell lines LN229, U138 and primary cells GBM#4, GBM#10 after normalization for 20,000, 25,000 and 30,000 cells. The raw values (A), median normed (B), median plus area normed (C), and median plus cell number normed (D) normalization approaches were used. Error bars correspond to the standard error of the mean. Stars depict statistically significant results with p < 0.05. The number of samples used in each group was as follows: nGBM10 15k = 13, nGBM10 20k = 16, nGBM10 25k = 13, nGBM4 15k = 12, nGBM4 20k = 15, nGBM4 25k = 17, nLN229 15k = 13, nLN229 20k = 14, nLN229 25k = 20, nU138 15k = 16, nU138 20k = 15, nU138 25k = 17. (TIF) [file pone.0347569.s005.TIF]

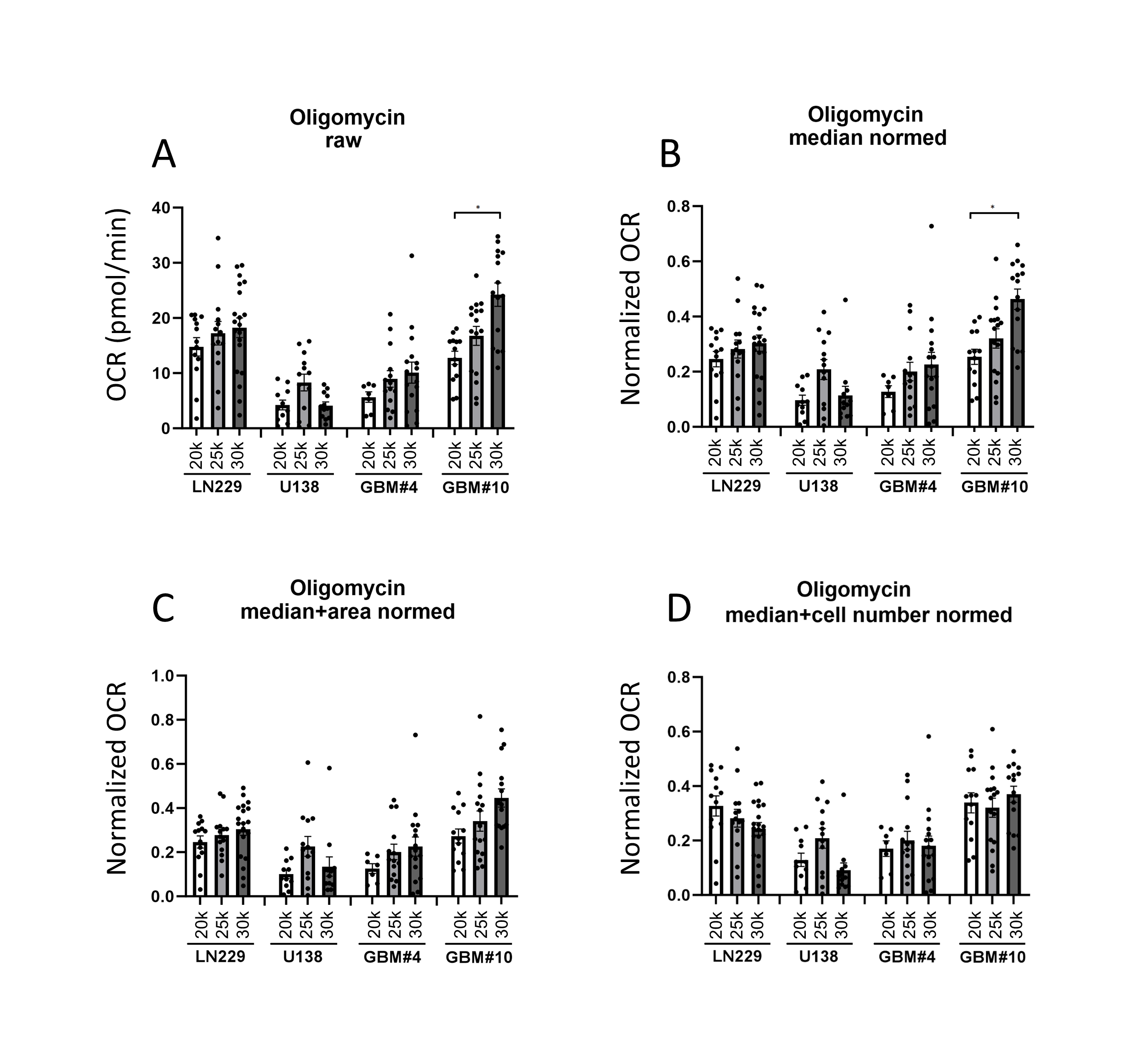

Supplement: S6 Fig — Oligomycin treatment compared between different glioblastoma cell lines LN229, U138 and primary cells GBM#4, GBM#10 after normalization for 20,000, 25,000 and 30,000 cells. The raw values (A), median normed (B), median plus area normed (C), and median plus cell number normed (D) normalization approaches were used. Error bars correspond to the standard error of the mean. Stars depict statistically significant results with p < 0.05. The number of samples used in each group was as follows: nGBM10 15k = 13, nGBM10 20k = 16, nGBM10 25k = 13, nGBM4 15k = 12, nGBM4 20k = 15, nGBM4 25k = 17, nLN229 15k = 13, nLN229 20k = 14, nLN229 25k = 20, nU138 15k = 16, nU138 20k = 15, nU138 25k = 17. (TIF) [file pone.0347569.s006.TIF]

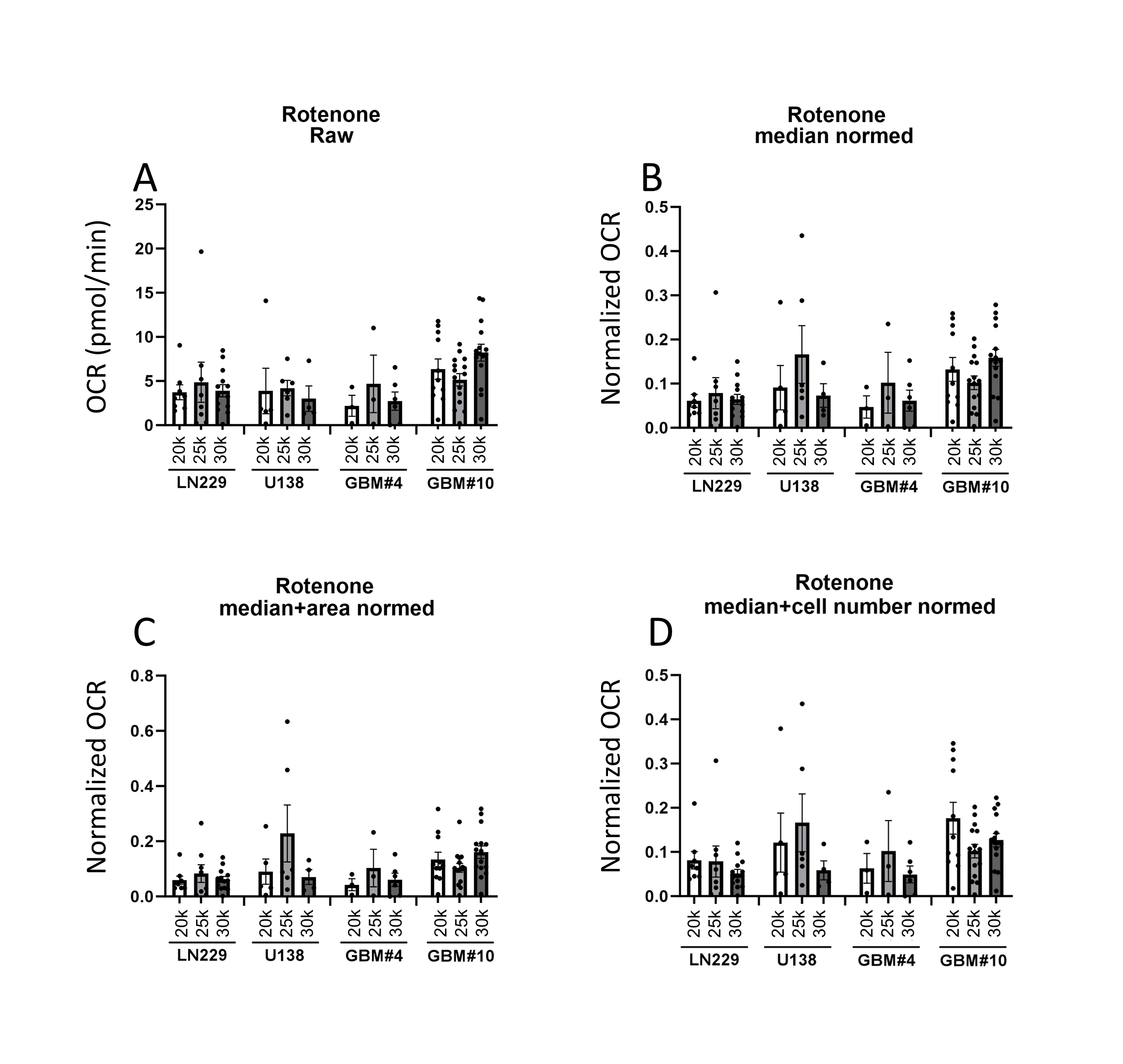

Supplement: S7 Fig — Rotenone plus antimycin A treatment compared between different glioblastoma cell lines LN229, U138 and primary cells GBM#4, GBM#10 after normalization for 20,000, 25,000 and 30,000 cells. The raw values (A), median normed (B), median plus area normed (C), and median plus cell number normed (D) normalization approaches were used. Error bars correspond to the standard error of the mean. Stars depict statistically significant results with p < 0.05. The number of samples used in each group was as follows: nGBM10 15k = 13, nGBM10 20k = 16, nGBM10 25k = 13, nGBM4 15k = 12, nGBM4 20k = 15, nGBM4 25k = 17, nLN229 15k = 13, nLN229 20k = 14, nLN229 25k = 20, nU138 15k = 16, nU138 20k = 15, nU138 25k = 17. (TIF) [file pone.0347569.s007.TIF]

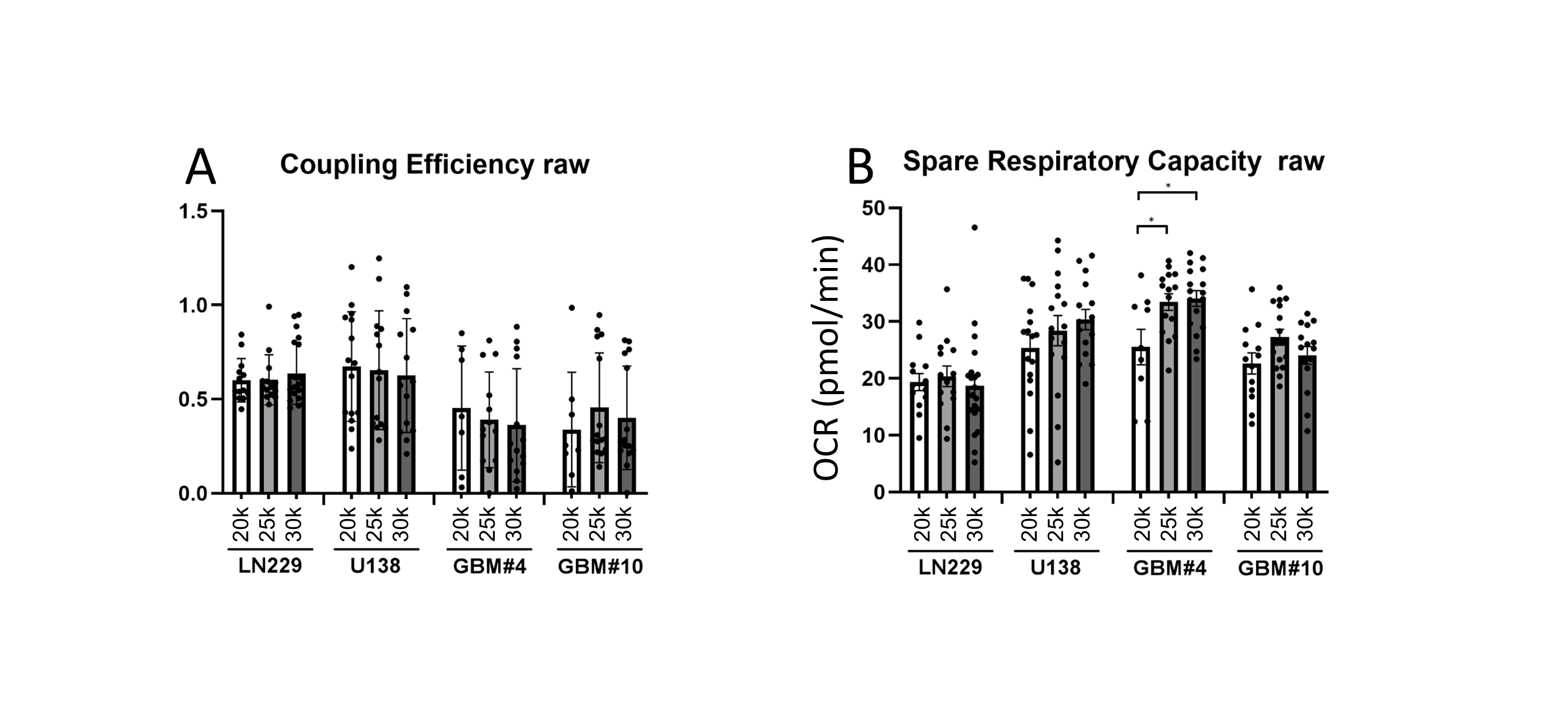

Supplement: S8 Fig — The raw values (A, B) of coupling efficiency and spare respiratory capacity compared between different glioblastoma cell lines LN229, U138 and primary cells GBM#4, GBM#10 after normalization for 20,000, 25,000 and 30,000 cells. The number of samples used in each group was as follows: nGBM10 15k = 13, nGBM10 20k = 16, nGBM10 25k = 13, nGBM4 15k = 12, nGBM4 20k = 15, nGBM4 25k = 17, nLN229 15k = 13, nLN229 20k = 14, nLN229 25k = 20, nU138 15k = 16, nU138 20k = 15, nU138 25k = 17. (TIF) [file pone.0347569.s008.TIF]

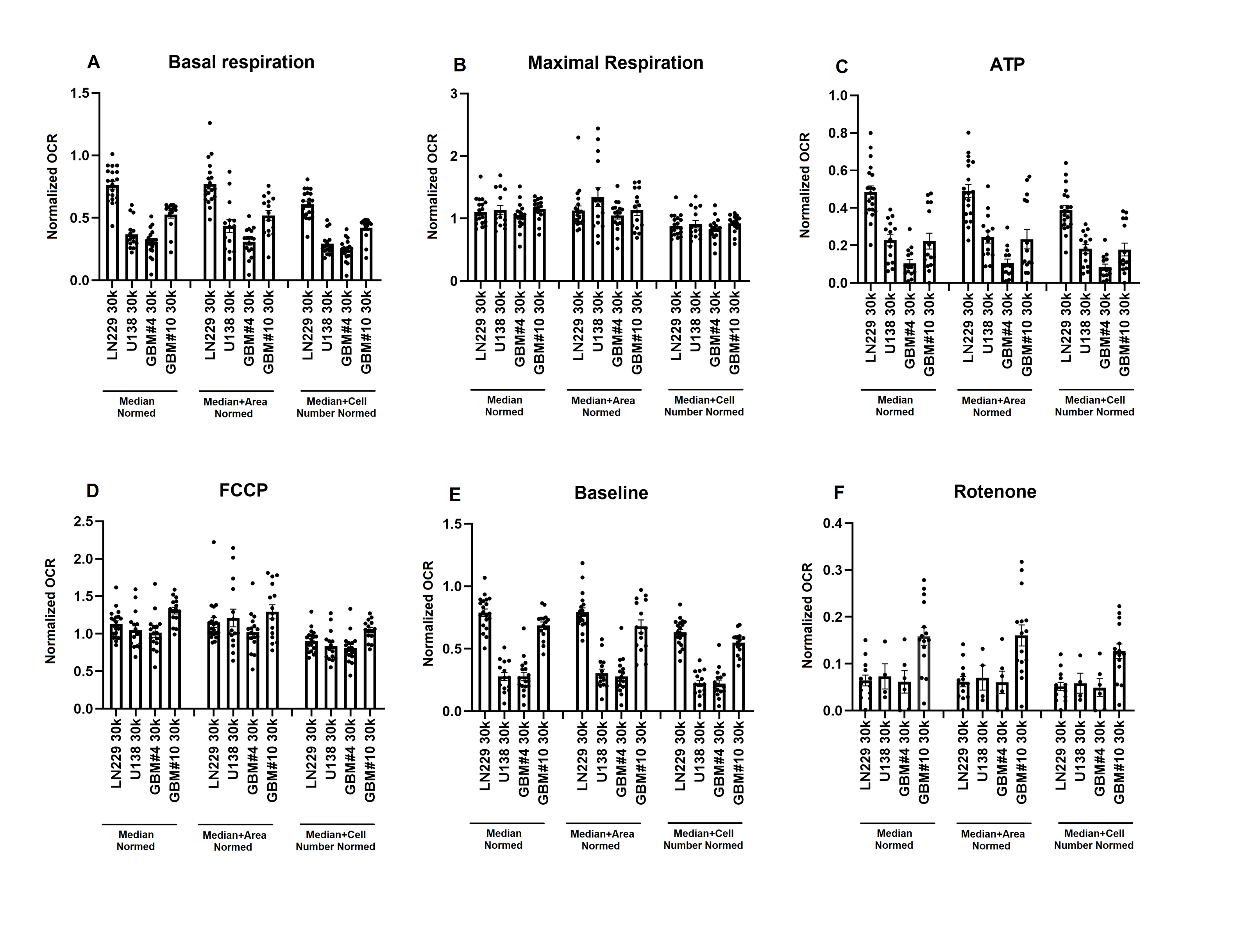

Supplement: S9 Fig — (A) Basal Respiration, (B) Maximal Respiration, (C) ATP, (D) FCCP, (E) Baseline and (F) Rotenone are shown. Error bars correspond to the standard error of the mean. The number of samples used in each group was as follows: nGBM10 15k = 13, nGBM10 20k = 16, nGBM10 25k = 13, nGBM4 15k = 12, nGBM4 20k = 15, nGBM4 25k = 17, nLN229 15k = 13, nLN229 20k = 14, nLN229 25k = 20, nU138 15k = 16, nU138 20k = 15, nU138 25k = 17. (TIF) [file pone.0347569.s009.TIF]

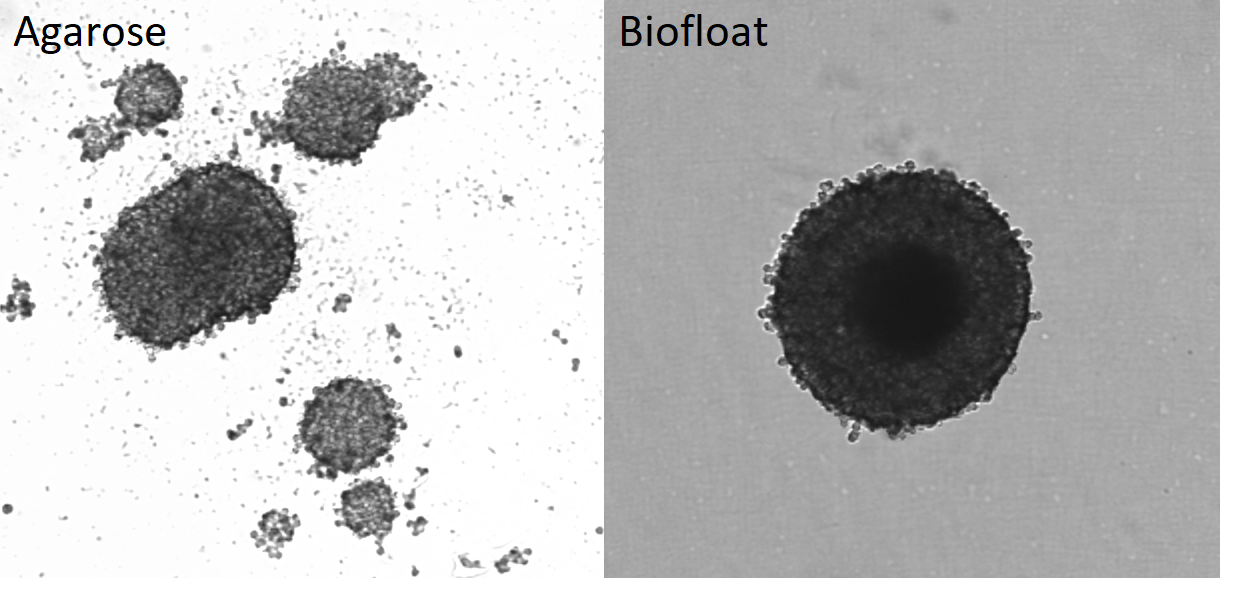

Supplement: S10 Fig — Example of LN229 spheroids generated with the agarose (left) and BioFloat (right) technique. Please denote the generation of multiple spheroids of different size with the agarose technique. Notably, all of those spheroids are significantly smaller compared to the case that all seeded cells formed exactly one spheroid (right image). (PNG) [file pone.0347569.s010.png]
