# Supplementary material for: An optimized protocol for metabolic measurement in 3D tumor spheroids derived from primary and established glioblastoma cells
Source: PLoS One. 2026 Apr 24;21(4):e0347569. doi: 10.1371/journal.pone.0347569 (PMC13108750; doi:10.1371/journal.pone.0347569)
Supplement: S1 Table — (DOCX) [file pone.0347569.s011.docx]

| Descriptive statistics of Basal Respiration Raw |  |  |  |  |  |  |  |  |  |  |  |  |  |  |  |
| --- | --- | --- | --- | --- | --- | --- | --- | --- | --- | --- | --- | --- | --- | --- | --- |
|  | LN229 15k | LN229 20k | LN229 25k |  | U138 15k | U138 20k | U138 25k |  | GBM#4 15k | GBM#4 20k | GBM#4 25k |  | GBM#10 15k | GBM  #10 20k | GBM  #10 25k |
| Number of values | 13 | 14 | 20 |  | 16 | 15 | 15 |  | 9 | 15 | 17 |  | 13 | 16 | 15 |
| Mean | 31,82 | 39,73 | 45,13 |  | 10,06 | 15,52 | 13,61 |  | 8,21 | 13,44 | 13,65 |  | 10,07 | 21,16 | 27,49 |
| Std. Error of Mean | 1,662 | 2,706 | 1,719 |  | 1,184 | 1,764 | 1,32 |  | 1,337 | 0,6342 | 1,191 |  | 1,189 | 1,23 | 1,608 |
| Descriptive statistics of Basal Respiration Raw Median Normed |  |  |  |  |  |  |  |  |  |  |  |  |  |  |  |
|  | LN229 15k | LN229 20k | LN229 25k |  | U138 15k | U138 20k | U138 25k |  | GBM#4 15k | GBM#4 20k | GBM#4 25k |  | GBM#10 15k | GBM#10 20k | GBM#10 25k |
| Number of values | 13 | 14 | 20 |  | 16 | 15 | 15 |  | 9 | 15 | 17 |  | 13 | 16 | 15 |
| Mean | 0,5324 | 0,6565 | 0,7623 |  | 0,2346 | 0,3559 | 0,3677 |  | 0,1852 | 0,2992 | 0,3071 |  | 0,194 | 0,4009 | 0,5271 |
| Std. Error of Mean | 0,0288 | 0,04454 | 0,03141 |  | 0,02256 | 0,03261 | 0,03 |  | 0,03137 | 0,01386 | 0,02705 |  | 0,02319 | 0,02099 | 0,0293 |
| Descriptive statistics of Basal Respiration Raw Median+Area Normed |  |  |  |  |  |  |  |  |  |  |  |  |  |  |  |
|  | LN229 15k | LN229 20k | LN229 25k |  | U138 15k | U138 20k | U138 25k |  | GBM#4 15k | GBM#4 20k | GBM#4 25k |  | GBM  #10 15k | GBM#10 20k | GBM#10 25k |
| Number of values | 13 | 14 | 20 |  | 16 | 15 | 15 |  | 9 | 15 | 17 |  | 13 | 16 | 15 |
| Mean | 0,5367 | 0,6995 | 0,7712 |  | 0,2436 | 0,3521 | 0,4341 |  | 0,1886 | 0,3002 | 0,3096 |  | 0,2243 | 0,4447 | 0,5184 |
| Std. Error of Mean | 0,034 | 0,07854 | 0,03893 |  | 0,02671 | 0,03225 | 0,05144 |  | 0,03277 | 0,01491 | 0,02759 |  | 0,03223 | 0,03881 | 0,04159 |
| Descriptive statistics of ATP production median+cell number normed korrigiert |  |  |  |  |  |  |  |  |  |  |  |  |  |  |  |
|  | LN229 15k | LN229 20k | LN229 25k |  | U138 15k | U138 20k | U138 25k |  | GBM#4 15k | GBM#4 20k | GBM#4 25k |  | GBM#10 15k | GBM#10 20k | GBM#10 25k |
| Number of values | 13 | 14 | 20 | 0 | 16 | 15 | 14 | 0 | 7 | 12 | 14 | 0 | 8 | 15 | 15 |
| Mean | 0,4191 | 0,3994 | 0,3878 |  | 0,2172 | 0,2031 | 0,1817 |  | 0,09045 | 0,1241 | 0,08354 |  | 0,1129 | 0,1872 | 0,1781 |
| Std. Error of Mean | 0,02399 | 0,04068 | 0,02598 |  | 0,02678 | 0,02292 | 0,02397 |  | 0,03361 | 0,01912 | 0,01648 |  | 0,0402 | 0,03245 | 0,03408 |
| Descriptive statistics of Basal Respiration Raw |  |  |  |  |  |  |  |  |  |  |  |  |  |  |  |
|  | LN229 15k | LN229 20k | LN229 25k |  | U138 15k | U138 20k | U138 25k |  | GBM#4 15k | GBM#4 20k | GBM#4 25k |  | GBM#10 15k | GBM#10 20k | GBM#10 25k |
| Number of values | 13 | 14 | 20 |  | 16 | 15 | 15 |  | 9 | 15 | 17 |  | 13 | 16 | 15 |
| Mean | 31,82 | 39,73 | 45,13 |  | 10,06 | 15,52 | 13,61 |  | 8,21 | 13,44 | 13,65 |  | 10,07 | 21,16 | 27,49 |
| Std. Error of Mean | 1,662 | 2,706 | 1,719 |  | 1,184 | 1,764 | 1,32 |  | 1,337 | 0,6342 | 1,191 |  | 1,189 | 1,23 | 1,608 |
| Descriptive statistics of Basal Respiration Raw Median Normed |  |  |  |  |  |  |  |  |  |  |  |  |  |  |  |
|  | LN229 15k | LN229 20k | LN229 25k |  | U138 15k | U138 20k | U138 25k |  | GBM#4 15k | GBM#4 20k | GBM#4 25k |  | GBM#10 15k | GBM#10 20k | GBM#10 25k |
| Number of values | 13 | 14 | 20 |  | 16 | 15 | 15 |  | 9 | 15 | 17 |  | 13 | 16 | 15 |
| Mean | 0,5324 | 0,6565 | 0,7623 |  | 0,2346 | 0,3559 | 0,3677 |  | 0,1852 | 0,2992 | 0,3071 |  | 0,194 | 0,4009 | 0,5271 |
| Std. Error of Mean | 0,0288 | 0,04454 | 0,03141 |  | 0,02256 | 0,03261 | 0,03 |  | 0,03137 | 0,01386 | 0,02705 |  | 0,02319 | 0,02099 | 0,0293 |
| Descriptive statistics of Basal Respiration Raw Median+Area Normed |  |  |  |  |  |  |  |  |  |  |  |  |  |  |  |
|  | LN229 15k | LN229 20k | LN229 25k |  | U138 15k | U138 20k | U138 25k |  | GBM#4 15k | GBM#4 20k | GBM#4 25k |  | GBM#10 15k | GBM#10 20k | GBM#10 25k |
| Number of values | 13 | 14 | 20 |  | 16 | 15 | 15 |  | 9 | 15 | 17 |  | 13 | 16 | 15 |
| Mean | 0,5367 | 0,6995 | 0,7712 |  | 0,2436 | 0,3521 | 0,4341 |  | 0,1886 | 0,3002 | 0,3096 |  | 0,2243 | 0,4447 | 0,5184 |
| Std. Error of Mean | 0,034 | 0,07854 | 0,03893 |  | 0,02671 | 0,03225 | 0,05144 |  | 0,03277 | 0,01491 | 0,02759 |  | 0,03223 | 0,03881 | 0,04159 |
| Descriptive statistics of Baseline Median+Cell Number Normed korrigiert |  |  |  |  |  |  |  |  |  |  |  |  |  |  |  |
|  | LN229 15k | LN229 20k | LN229 25k |  | U138 15k | U138 20k | U138 25k |  | GBM#4 15k | GBM#4 20k | GBM#4 25k |  | GBM#10 15k | GBM  #10 20k | GBM#10 25k |
| Number of values | 13 | 14 | 20 |  | 16 | 16 | 15 | 0 | 9 | 15 | 17 |  | 13 | 16 | 15 |
| Mean | 0,7466 | 0,6817 | 0,6308 |  | 0,2629 | 0,3517 | 0,2228 |  | 0,1984 | 0,2625 | 0,2224 |  | 0,3985 | 0,4957 | 0,5484 |
| Std. Error of Mean | 0,04376 | 0,05034 | 0,02379 |  | 0,03404 | 0,03451 | 0,02512 |  | 0,04148 | 0,02679 | 0,02727 |  | 0,04101 | 0,02046 | 0,02336 |
| Descriptive statistics of ATP production raw | |  |  |  |  |  |  |  |  |  |  |  |  |  |  |
|  | LN229 15k | LN229 20k | LN229 25k |  | U138 15k | U138 20k | U138 25k |  | GBM#4 15k | GBM#4 20k | GBM#4 25k |  | GBM#10 15k | GBM#10 20k | GBM#10 25k |
| Number of values | 13 | 14 | 20 |  | 16 | 15 | 14 |  | 7 | 12 | 14 |  | 8 | 15 | 15 |
| Mean | 18,77 | 24,07 | 28,54 |  | 7,02 | 8,499 | 9,063 |  | 3,049 | 5,671 | 4,684 |  | 4,246 | 9,805 | 11,46 |
| Std. Error of Mean | 1,002 | 2,301 | 1,709 |  | 1,043 | 1,365 | 1,535 |  | 1,127 | 0,8745 | 0,9441 |  | 1,473 | 1,622 | 2,161 |
| Descriptive statistics of ATP production median normed |  |  |  |  |  |  |  |  |  |  |  |  |  |  |  |
|  | LN229 15k | LN229 20k | LN229 25k |  | U138 15k | U138 20k | U138 25k |  | GBM#4 15k | GBM#4 20k | GBM#4 25k |  | GBM#10 15k | GBM#10 20k | GBM#10 25k |
| Number of values | 13 | 14 | 20 |  | 16 | 15 | 14 |  | 7 | 12 | 14 |  | 8 | 15 | 15 |
| Mean | 0,3143 | 0,3994 | 0,4848 |  | 0,1629 | 0,2031 | 0,2271 |  | 0,06784 | 0,1241 | 0,1044 |  | 0,08468 | 0,1872 | 0,2227 |
| Std. Error of Mean | 0,01799 | 0,04068 | 0,03247 |  | 0,02009 | 0,02292 | 0,02997 |  | 0,0252 | 0,01912 | 0,0206 |  | 0,03015 | 0,03245 | 0,04259 |
| Descriptive statistics of ATP production median+area normed |  |  |  |  |  |  |  |  |  |  |  |  |  |  |  |
|  | LN229 15k | LN229 20k | LN229 25k |  | U138 15k | U138 20k | U138 25k |  | GBM#4 15k | GBM#4 20k | GBM#4 25k |  | GBM#10 15k | GBM#10 20k | GBM#10 25k |
| Number of values | 13 | 14 | 20 |  | 16 | 15 | 14 |  | 7 | 12 | 14 |  | 8 | 15 | 15 |
| Mean | 0,3165 | 0,451 | 0,49 |  | 0,1657 | 0,2059 | 0,2445 |  | 0,07082 | 0,1246 | 0,1061 |  | 0,09572 | 0,2134 | 0,233 |
| Std. Error of Mean | 0,01967 | 0,0897 | 0,03421 |  | 0,0213 | 0,02096 | 0,03277 |  | 0,02869 | 0,02069 | 0,02106 |  | 0,03264 | 0,03814 | 0,05142 |
| Descriptive statistics of ATP production median+cell number normed korrigiert |  |  |  |  |  |  |  |  |  |  |  |  |  |  |  |
|  | LN229 15k | LN229 20k | LN229 25k |  | U138 15k | U138 20k | U138 25k |  | GBM#4 15k | GBM#4 20k | GBM#4 25k |  | GBM#10 15k | GBM#10 20k | GBM#10 25k |
| Number of values | 13 | 14 | 20 |  | 16 | 15 | 14 | 0 | 7 | 12 | 14 |  | 8 | 15 | 15 |
| Mean | 0,4191 | 0,3994 | 0,3878 |  | 0,2172 | 0,2031 | 0,1817 |  | 0,09045 | 0,1241 | 0,08354 |  | 0,1129 | 0,1872 | 0,1781 |
| Std. Error of Mean | 0,02399 | 0,04068 | 0,02598 |  | 0,02678 | 0,02292 | 0,02397 |  | 0,03361 | 0,01912 | 0,01648 |  | 0,0402 | 0,03245 | 0,03408 |
| Descriptive statistics of FCCP Raw | | |  |  |  |  |  |  |  |  |  |  |  |  |  |
|  | LN229 15k | LN229 20k | LN229 25k |  | U138 15k | U138 20k | U138 25k |  | GBM#4 15k | GBM#4 20k | GBM#4 25k |  | GBM#10 15k | GBM#10 20k | GBM#10 25k |
| Number of values | 13 | 14 | 20 |  | 16 | 16 | 15 |  | 9 | 15 | 17 |  | 13 | 16 | 15 |
| Mean | 54,64 | 63,31 | 67,09 |  | 32,53 | 42,57 | 39,05 |  | 30,59 | 43,68 | 45,19 |  | 42,62 | 57,97 | 67,96 |
| Std. Error of Mean | 2,873 | 3,415 | 2,425 |  | 2,258 | 3,247 | 3,392 |  | 2,82 | 1,98 | 2,594 |  | 1,893 | 1,396 | 2,337 |
| Descriptive statistics of FCCP Median Normed |  |  |  |  |  |  |  |  |  |  |  |  |  |  |  |
|  | LN229 15k | LN229 20k | LN229 25k |  | U138 15k | U138 20k | U138 25k |  | GBM#4 15k | GBM#4 20k | GBM#4 25k |  | GBM#10 15k | GBM#10 20k | GBM#10 25k |
| Number of values | 13 | 14 | 20 |  | 16 | 16 | 15 |  | 9 | 15 | 17 |  | 13 | 16 | 15 |
| Mean | 0,9123 | 1,043 | 1,13 |  | 0,7617 | 1,057 | 1,047 |  | 0,6861 | 0,9716 | 1,013 |  | 0,8409 | 1,108 | 1,308 |
| Std. Error of Mean | 0,04544 | 0,05155 | 0,04118 |  | 0,03597 | 0,04437 | 0,06661 |  | 0,0667 | 0,04063 | 0,05904 |  | 0,05233 | 0,03108 | 0,04494 |
| Descriptive statistics of FCCP Median+Area Normed |  |  |  |  |  |  |  |  |  |  |  |  |  |  |  |
|  | LN229 15k | LN229 20k | LN229 25k |  | U138 15k | U138 20k | U138 25k |  | GBM#4 15k | GBM#4 20k | GBM#4 25k |  | GBM#10 15k | GBM#10 20k | GBM#10 25k |
| Number of values | 13 | 14 | 20 |  | 16 | 16 | 15 |  | 9 | 15 | 17 |  | 13 | 16 | 15 |
| Mean | 0,9184 | 1,111 | 1,151 |  | 0,787 | 1,118 | 1,211 |  | 0,6947 | 0,9763 | 1,018 |  | 0,9246 | 1,206 | 1,293 |
| Std. Error of Mean | 0,05125 | 0,1215 | 0,06557 |  | 0,06016 | 0,09571 | 0,1181 |  | 0,07515 | 0,04424 | 0,06147 |  | 0,07733 | 0,08529 | 0,09304 |
| FCCP Median+Cell Number Normed korrigiert |  |  |  |  |  |  |  |  |  |  |  |  |  |  |  |
|  | LN229 15k | LN229 20k | LN229 25k |  | U138 15k | U138 20k | U138 25k |  | GBM#4 15k | GBM#4 20k | GBM#4 25k |  | GBM#10 15k | GBM#10 20k | GBM#10 25k |
| Number of values | 13 | 14 | 20 |  | 16 | 16 | 15 |  | 9 | 15 | 17 |  | 13 | 16 | 15 |
| Mean | 1,216 | 1,043 | 0,904 |  | 1,016 | 1,057 | 0,8372 |  | 0,9148 | 0,9716 | 0,8105 |  | 1,121 | 1,108 | 1,047 |
| Std. Error of Mean | 0,06059 | 0,05155 | 0,03294 |  | 0,04796 | 0,04437 | 0,05329 |  | 0,08893 | 0,04063 | 0,04723 |  | 0,06977 | 0,03108 | 0,03595 |
| Descriptive statistics of MaxRespiration Raw |  |  |  |  |  |  |  |  |  |  |  |  |  |  |  |
|  | LN229 15k | LN229 20k | LN229 25k |  | U138 15k | U138 20k | U138 25k |  | GBM#4 15k | GBM#4 20k | GBM#4 25k |  | GBM#10 15k | GBM#10 20k | GBM#10 25k |
| Number of values | 13 | 14 | 20 |  | 16 | 16 | 15 |  | 9 | 15 | 17 |  | 13 | 16 | 15 |
| Mean | 52,91 | 61,72 | 65,45 |  | 33,99 | 42,74 | 41,51 |  | 32,16 | 45,28 | 46,45 |  | 37,66 | 53,19 | 59,75 |
| Std. Error of Mean | 2,56 | 2,892 | 2,61 |  | 2,51 | 3,675 | 2,997 |  | 3,377 | 1,532 | 2,3 |  | 1,734 | 1,828 | 2,404 |
| Descriptive statistics of MaxRespiration Median Normed |  |  |  |  |  |  |  |  |  |  |  |  |  |  |  |
|  | LN229 15k | LN229 20k | LN229 25k |  | U138 15k | U138 20k | U138 25k |  | GBM#4 15k | GBM#4 20k | GBM#4 25k |  | GBM#10 15k | GBM#10 20k | GBM#10 25k |
| Number of values | 13 | 14 | 20 |  | 16 | 16 | 15 |  | 9 | 15 | 17 |  | 13 | 16 | 15 |
| Mean | 0,8847 | 1,017 | 1,104 |  | 0,7991 | 1,037 | 1,136 |  | 0,7225 | 1,008 | 1,042 |  | 0,736 | 1,013 | 1,15 |
| Std. Error of Mean | 0,04212 | 0,04349 | 0,04634 |  | 0,04293 | 0,04477 | 0,07455 |  | 0,07996 | 0,03249 | 0,05268 |  | 0,03606 | 0,03119 | 0,04586 |
| Descriptive statistics of MaxRespiration Raw Median+Area Normed |  |  |  |  |  |  |  |  |  |  |  |  |  |  |  |
|  | LN229 15k | LN229 20k | LN229 25k |  | U138 15k | U138 20k | U138 25k |  | GBM#4 15k | GBM#4 20k | GBM#4 25k |  | GBM#10 15k | GBM#10 20k | GBM#10 25k |
| Number of values | 13 | 14 | 20 |  | 16 | 16 | 15 |  | 9 | 15 | 17 |  | 13 | 16 | 15 |
| Mean | 0,8925 | 1,083 | 1,128 |  | 0,8297 | 1,075 | 1,341 |  | 0,7337 | 1,013 | 1,049 |  | 0,8228 | 1,109 | 1,133 |
| Std. Error of Mean | 0,05154 | 0,1117 | 0,07313 |  | 0,06958 | 0,06794 | 0,1489 |  | 0,08841 | 0,03755 | 0,05707 |  | 0,07593 | 0,08194 | 0,08249 |
| Max Respiration Raw Median+Cell Number Normed korr |  |  |  |  |  |  |  |  |  |  |  |  |  |  |  |
|  | LN229 15k | LN229 20k | LN229 25k |  | U138 15k | U138 20k | U138 25k |  | GBM#4 15k | GBM#4 20k | GBM#4 25k |  | GBM#10 15k | GBM#10 20k | GBM#10 25k |
| Number of values | 13 | 14 | 20 | 0 | 16 | 16 | 15 | 0 | 9 | 15 | 17 | 0 | 13 | 16 | 15 |
| Mean | 1,18 | 1,017 | 0,883 |  | 1,066 | 1,037 | 0,9086 |  | 0,9634 | 1,008 | 0,8338 |  | 0,9814 | 1,013 | 0,9201 |
| Std. Error of Mean | 0,05616 | 0,04349 | 0,03708 |  | 0,05724 | 0,04477 | 0,05964 |  | 0,1066 | 0,03249 | 0,04214 |  | 0,04808 | 0,03119 | 0,03669 |
| Descriptive statistics of Oligomycin raw |  |  |  |  |  |  |  |  |  |  |  |  |  |  |  |
|  | LN229 15k | LN229 20k | LN229 25k |  | U138 15k | U138 20k | U138 25k |  | GBM#4 15k | GBM#4 20k | GBM#4 25k |  | GBM#10 15k | GBM#10 20k | GBM#10 25k |
| Number of values | 13 | 14 | 20 |  | 11 | 13 | 12 |  | 7 | 14 | 16 |  | 13 | 16 | 15 |
| Mean | 14,78 | 17,25 | 18,23 |  | 4,235 | 8,316 | 4,105 |  | 5,666 | 8,947 | 10,09 |  | 12,77 | 16,76 | 24,24 |
| Std. Error of Mean | 1,669 | 2,123 | 1,793 |  | 0,894 | 1,517 | 0,6786 |  | 0,9342 | 1,501 | 1,912 |  | 1,2 | 1,726 | 2,112 |
| Descriptive statistics of Oligomycin median normed |  |  |  |  |  |  |  |  |  |  |  |  |  |  |  |
|  | LN229 15k | LN229 20k | LN229 25k |  | U138 15k | U138 20k | U138 25k |  | GBM#4 15k | GBM#4 20k | GBM#4 25k |  | GBM#10 15k | GBM#10 20k | GBM#10 25k |
| Number of values | 13 | 14 | 20 |  | 11 | 13 | 12 |  | 7 | 14 | 16 |  | 13 | 16 | 15 |
| Mean | 0,2457 | 0,2824 | 0,3037 |  | 0,09685 | 0,2081 | 0,1138 |  | 0,1276 | 0,2005 | 0,2265 |  | 0,254 | 0,3207 | 0,4628 |
| Std. Error of Mean | 0,02776 | 0,03265 | 0,02916 |  | 0,01824 | 0,03639 | 0,03353 |  | 0,02159 | 0,03376 | 0,04357 |  | 0,02754 | 0,03495 | 0,03703 |
| Descriptive statistics of Oligomycin median+area normed |  |  |  |  |  |  |  |  |  |  |  |  |  |  |  |
|  | LN229 15k | LN229 20k | LN229 25k |  | U138 15k | U138 20k | U138 25k |  | GBM#4 15k | GBM#4 20k | GBM#4 25k |  | GBM#10 15k | GBM#10 20k | GBM#10 25k |
| Number of values | 13 | 14 | 20 |  | 11 | 13 | 12 |  | 7 | 14 | 16 |  | 13 | 16 | 15 |
| Mean | 0,2461 | 0,277 | 0,3041 |  | 0,1 | 0,2261 | 0,1348 |  | 0,1257 | 0,2013 | 0,2253 |  | 0,2731 | 0,3413 | 0,4456 |
| Std. Error of Mean | 0,0274 | 0,02692 | 0,02793 |  | 0,01978 | 0,04495 | 0,04373 |  | 0,02187 | 0,03497 | 0,04347 |  | 0,0321 | 0,04594 | 0,04106 |
| Oligomycin median+cell number normed korr |  |  |  |  |  |  |  |  |  |  |  |  |  |  |  |
|  | LN229 15k | LN229 20k | LN229 25k |  | U138 15k | U138 20k | U138 25k |  | GBM#4 15k | GBM#4 20k | GBM#4 25k |  | GBM#10 15k | GBM#10 20k | GBM#10 25k |
| Number of values | 13 | 14 | 20 | 0 | 11 | 13 | 12 | 0 | 7 | 14 | 16 | 0 | 13 | 16 | 15 |
| Mean | 0,3276 | 0,2824 | 0,243 |  | 0,1291 | 0,2081 | 0,09103 |  | 0,1702 | 0,2005 | 0,1812 |  | 0,3387 | 0,3207 | 0,3702 |
| Std. Error of Mean | 0,03702 | 0,03265 | 0,02333 |  | 0,02432 | 0,03639 | 0,02683 |  | 0,02878 | 0,03376 | 0,03486 |  | 0,03672 | 0,03495 | 0,02963 |
| Descriptive statistics of Rotenone Raw |  |  |  |  |  |  |  |  |  |  |  |  |  |  |  |
|  | LN229 15k | LN229 20k | LN229 25k |  | U138 15k | U138 20k | U138 25k |  | GBM#4 15k | GBM#4 20k | GBM#4 25k |  | GBM#10 15k | GBM#10 20k | GBM#10 25k |
| Number of values | 8 | 8 | 13 |  | 5 | 6 | 4 |  | 3 | 3 | 6 |  | 11 | 15 | 15 |
| Mean | 3,726 | 4,867 | 3,9 |  | 3,881 | 4,203 | 3,007 |  | 2,198 | 4,695 | 2,732 |  | 6,35 | 5,14 | 8,214 |
| Std. Error of Mean | 0,8471 | 2,266 | 0,6868 |  | 2,569 | 0,8785 | 1,441 |  | 1,183 | 3,26 | 1,036 |  | 1,155 | 0,6991 | 0,9564 |
| Descriptive statistics of Rotenone median normed |  |  |  |  |  |  |  |  |  |  |  |  |  |  |  |
|  | LN229 15k | LN229 20k | LN229 25k |  | U138 15k | U138 20k | U138 25k |  | GBM#4 15k | GBM#4 20k | GBM#4 25k |  | GBM#10 15k | GBM#10 20k | GBM#10 25k |
| Number of values | 8 | 8 | 13 |  | 5 | 6 | 4 |  | 3 | 3 | 6 |  | 11 | 15 | 15 |
| Mean | 0,06101 | 0,0784 | 0,06441 |  | 0,09094 | 0,166 | 0,07312 |  | 0,04708 | 0,1021 | 0,06136 |  | 0,1323 | 0,1018 | 0,1584 |
| Std. Error of Mean | 0,01479 | 0,03528 | 0,01142 |  | 0,05028 | 0,06544 | 0,0268 |  | 0,02514 | 0,06911 | 0,02373 |  | 0,02704 | 0,01535 | 0,01924 |
| Descriptive statistics of Rotenone median+area normed | |  |  |  |  |  |  |  |  |  |  |  |  |  |  |
|  | LN229 15k | LN229 20k | LN229 25k |  | U138 15k | U138 20k | U138 25k |  | GBM#4 15k | GBM#4 20k | GBM#4 25k |  | GBM#10 15k | GBM#10 20k | GBM#10 25k |
| Number of values | 8 | 8 | 13 |  | 5 | 6 | 4 |  | 3 | 3 | 6 |  | 11 | 15 | 15 |
| Mean | 0,05904 | 0,08311 | 0,06172 |  | 0,08983 | 0,2284 | 0,07032 |  | 0,04263 | 0,103 | 0,06033 |  | 0,1334 | 0,1036 | 0,1602 |
| Std. Error of Mean | 0,01443 | 0,03196 | 0,01077 |  | 0,04554 | 0,1035 | 0,02638 |  | 0,02165 | 0,06786 | 0,02369 |  | 0,0266 | 0,01609 | 0,02251 |
| Max Respiration Raw Median+Cell Number Normed korr |  |  |  |  |  |  |  |  |  |  |  |  |  |  |  |
|  | LN229 15k | LN229 20k | LN229 25k |  | U138 15k | U138 20k | U138 25k |  | GBM#4 15k | GBM#4 20k | GBM#4 25k |  | GBM#10 15k | GBM#10 20k | GBM#10 25k |
| Number of values | 13 | 14 | 20 |  | 16 | 16 | 15 |  | 9 | 15 | 17 |  | 13 | 16 | 15 |
| Mean | 1,18 | 1,017 | 0,883 |  | 1,066 | 1,037 | 0,9086 |  | 0,9634 | 1,008 | 0,8338 |  | 0,9814 | 1,013 | 0,9201 |
| Std. Error of Mean | 0,05616 | 0,04349 | 0,03708 |  | 0,05724 | 0,04477 | 0,05964 |  | 0,1066 | 0,03249 | 0,04214 |  | 0,04808 | 0,03119 | 0,03669 |
